# *Antirhea borbonica* Aqueous Extract Protects Albumin and Erythrocytes from Glycoxidative Damages

**DOI:** 10.3390/antiox9050415

**Published:** 2020-05-12

**Authors:** Jade Delveaux, Chloé Turpin, Bryan Veeren, Nicolas Diotel, Susana B. Bravo, Floran Begue, Ezequiel Álvarez, Olivier Meilhac, Emmanuel Bourdon, Philippe Rondeau

**Affiliations:** 1 Université de La Réunion, INSERM, UMR 1188 Diabète athérothombose Thérapies Réunion Océan Indien (DéTROI), 97400 Saint-Denis de La Réunion, France; jade.delveaux@gmail.com (J.D.); tur.chloe@gmail.com (C.T.); veeren.b@hotmail.fr (B.V.); nicolas.diotel@univ-reunion.fr (N.D.); begue.floran@hotmail.fr (F.B.); olivier.meilhac@inserm.fr (O.M.); 2 Proteomic Unit and Instituto de Investigación Sanitaria de Santiago de Compostela (IDIS), Complexo Hospitalario Universitario de Santiago de Compostela (CHUS), SERGAS, 15706 Santiago de Compostela, Spain; sbbravo@gmail.com (S.B.B.); ezequiel.alvarez.castro@gmail.com (E.Á.); 3 CIBERCV, 28029 Madrid, Spain; 4 Centre hospitalier universitaire de La Réunion, 97400 Saint Denis, France

**Keywords:** *Antirhea borbonica*, albumin, erythrocytes, diabetes, glycation, antioxidant, oxidative stress, zebrafish

## Abstract

Diabetes constitutes a major health problem associated with severe complications. In hyperglycemic conditions, chronically increased oxidation and glycation of circulating components lead to advanced glycation end-products (AGEs) formation, a key contributor in diabetes complication progression. In line with literature documenting the beneficial properties of herbal teas, this study evaluates the antioxidant/glycant properties of *Antirhea borbonica* (*Ab*). *Ab* aqueous extract effects were tested on human albumin or erythrocytes submitted to methyl glyoxal-mediated glycoxidative damages. By using mass spectrometry, *Ab* aqueous extracts revealed to be rich in polyphenols. All tested biomarkers of oxidation and glycation, such as AGE, ketoamine, oxidized thiol groups, were decreased in albumin when glycated in the presence of *Ab* aqueous extract. *Ab* extract preserve erythrocyte from methylglyoxal (MGO)-induced damages in terms of restored membrane deformability, reduced oxidative stress and eryptosis phenomenon. Antioxidant capacities of *Ab* extract on erythrocytes were retrieved in vivo in zebrafish previously infused with MGO. These results bring new evidences on the deleterious impacts of glycation on albumin and erythrocyte in diabetes. Furthermore, it reveals antioxidant and antiglycant properties of *Ab* that could be used for the dietary modulation of oxidative stress and glycation in hyperglycemic situations.

## 1. Introduction

Diabetes pathology is associated with hyperglycemia and an enhanced risk of developing cardiovascular disease [1]. Several metabolic and signaling pathways become dysfunctional and contribute to chronic disorders associated with diabetes. For instance, oxidative stress and glycation leading to advanced glycation end-products (AGEs) formation, were identified as key mediators of multiple complications occurring in diabetes [2]. The generation of AGEs results from a complex process, named glycoxidation and involves a condensation reaction between proteins and glucose, followed by Amadori rearrangement, cyclization, polymerization, cleavage and oxidation process [3]. Glycoxidation process can also concern protein reaction with highly reactive α-oxoaldehydes derivatives such as methylglyoxal (MGO) occurring in glucose autoxidation [4]; AGEs derived from these precursors are responsible for several organ and cell toxicities [5]. Plasmatic proteins and blood cells are the major targets of oxidation and glycation affecting mainly long half-life and abundant blood proteins such as albumin and hemoglobin [6]. Albumin was considered as a preferential protein target for glycoxidation because of its long half-life, its high abundance in serum (35 g/L), its continuous exposure to oxidative stress and hyperglycemia and the presence of multiple glycation sites [6]. The glycoxidation process of this globular protein exerts adverse effects on its structural and functional integrity, particularly in terms of conformational changes and redox status, which may favor the formation of cross β-structure rich amyloid fibril [7,8]. Our group recently evidenced impaired drug binding capacity of albumin when glycoxidized [9,10]. Glycoxidized albumin was associated with metabolic disorders observed in diabetes mellitus, such as retinopathy, nephropathy and coronary artery disease [11].

During their life span, erythrocytes are also continuously exposed to glucose and oxidative stress. In hyperglycemic condition, red blood cells are particularly concerned by hemoglobin glycation and AGEs abundant accumulations [12]. In red blood cells, glycoxidation phenomenon can lead to an increased oxidative stress, aggregation, membrane phospholipids asymmetry resulting from eryptosis and to a decreased deformability and elasticity capacity [13]. These hemorrheologic impairments, associated with an induced early senescence phenomenon, render red blood cells (RBC) more prone to being phagocytized and potentially participate in atheroma/vascular complications [14,15,16]. 

Among the potential therapeutic interventions to manage diabetes and prevent for associated complications, polyphenol-rich diet from natural products appears to be a high interest approach [17]. Plant polyphenols constitute the main dietary source of antioxidants which exhibit several other beneficial biological properties including antibacterial, antiviral and anti-inflammatory activities [18,19].

Endemic to Mascarene and Madagascar, *Antirhea borbonica* (Rubiaceae) is a tree mainly found in the wild and humid forests of Reunion island, a French overseas territory. Formerly used in Reunion, the leaves were crushed and applied in poultices to improve healing and stop bleeding or consumed as a decoction for their astringent properties to fight against diarrhea, dysentery and bladder problems [20]. Registered in the French Pharmacopoeia, this protected species is cultivated and sold (≈16 €/100 g) on a small scale mainly at La Reunion island [20]. Today, leaf decoctions remain consumed by Mauritians for their astringent properties [21,22] while the Reunionese use it in the form of herbal teas to treat diabetes mellitus and hypercholesterolemia [23].

Recently, published data from our group evidenced polyphenols derived from the medicinal plant *Antirhea borbonica* exhibit robust anti-inflammatory properties at the preadipocyte and adipocyte levels [24,25]. The benefits of this plant in terms of antioxidative and antiglycative properties remained uncovered. In this study, we hypothesized that the *Antirhea borbonica* (*Ab*) aqueous plant extracts exert antiglycation and antioxidant protective effects for albumin and RBC subjected to dicarbonyl-induced toxicity. For albumin, the main plasmatic protein, multistage glycation markers (ketoamine, free amine level and fluorescent AGE), along with oxidative and conformational parameters (thiols, β-aggregation and tryptophan fluorescence), were investigated. For the main circulating blood cells, morphology, deformability, oxidative stress and eryptosis parameters were evaluated. Finally, the antioxidant effects of *Ab* in red blood cells were also investigated in an in vivo model of MGO-injected zebrafish.

In summary, *Ab* aqueous extracts revealed to be rich in polyphenols and potent inhibitor of oxidative damages for albumin and erythrocytes submitted to MGO-induced damages. Antioxidant capacities of *Ab* extract on erythrocytes were retrieved in vivo in zebrafish previously infused with MGO. These results reveal antioxidant and antiglycant properties of *Ab* that could be used for dietary modulation of oxidative stress and glycation in hyperglycemic situations.

## 2. Materials and Methods 

### 2.1. Plant Materials and Preparation of Plant Extracts

Leaves of *Antirhea borbonica* J.F Gmel (Bois d’osto; Saint-Joseph de La Réunion; REF: BOSJDTCA171218AA) obtained from the Cooperative des Huiles Essentielles de Bourbon (CAHEB) (Saint-Pierre de La Réunion, France) were dried, crushed and conserved at –20 °C until extraction. Aqueous plant extract (or herbal tea) (4 g/L) was prepared by infusion technique. Briefly, 1 g of crushed plant was mixed with 250 mL of boiled Milli-Q water (or fish water) for 10 min. The herbal tea preparation was filtered (with 20 µm membrane), aliquoted and stored at –80 °C before use. For zebrafish treatment, the fresh herbal tea was directly diluted in 2 L of fish water to reach a final concentration of 0.5 g/L. 

### 2.2. Identification of Polyphenols in Medicinal Plant Extract

Polyphenols extracted from *Antirhea borbonica* infusion were identified by Ultra-High Performance Liquid Chromatography coupled with diode array detection and HESI-Orbitrap mass spectrometer (Q Exactive Plus, Thermo Fisher, Waltham, MA, United States). Briefly, 10 µL of sample was injected using an UHPLC system equipped with a Thermo Fisher Ultimate 3000 series WPS-3000 RS autosampler and then separated on a PFP column (2.6 µm, 100 mm × 2.1 mm, Phenomenex, Torrance, CA, United States). Elution of the column was conducted by using a gradient combination of 0.1% formic acid in water (A) and 0.1% formic acid in acetonitrile (B) at the flow rate of 0.450 mL/min, with 5% B at 0–0.1 min, 35% B at 0.1–7.1 min, 95% B at 7.1–7.2 min, 95% B at 7.2–7.9 min, 5% B at 7.9–8 min and 5% B at 8–10 min. The column temperature was held at 30 °C and the detection wavelength was set to 280 nm and 320 nm. 

For the mass spectrometer conditions, a Heated Electrospray Ionization Source II (HESI II) was used. Nitrogen was used as drying gas. The mass spectrometric conditions were optimized as follows—spray voltage = 2.8 kV, capillary temperature = 350 °C, sheath gas flow rate = 60 units, aux gas flow rate = 20 units and S lens RF level = 50. 

Mass spectra were registered in full scan mode from *m/z* 100 to 1500 in negative ion mode at a resolving power of 70,000 FWHM (full width at half maximum) at *m/z* 400. The automatic gain control (AGC) was set at 1e6. The Orbitrap performance in negative ionization mode was evaluated weekly and external calibration of the mass spectrometer was performed with a LTQ ESI negative ion calibration solution (Pierce™, Waltham, MA, United States). Identification of the compounds of interest was based on their exact mass, retention time and MS/MS analysis. Data were acquired and processed by XCalibur 4.0 software (Thermo Fisher Scientific Inc., Waltham, MA, United States).

### 2.3. Quantification and Identification of Polyphenols in Medicinal Plant Extracts

Polyphenol contents in herbal tea preparation of *Antirhea borbonica* were determined using the Folin-Ciocalteu test [26]. Briefly, 25 μL plant extract (1:10 dilution), 125 μL Folin-Ciocalteu’s phenol reagent (Sigma-Aldrich, Darmstadt, Germany, 1:10 dilution in water) and 100 μL sodium carbonate solution (75 g/L) were mixed in a ninety six-well microplate and sequentially incubated at 50 °C for 5 min and then at 4 °C for 5 min, in the dark before absorbance measurement at 760 nm (FLUOstar Optima, BMG Labtech, Ortenberg, Germany). The total phenolic content was calculated with respect to the gallic acid (Sigma-Aldrich, Darmstadt, Germany) calibration curve (calibration range 12.5–300 µM). Results were expressed as mg gallic acid equivalent (GAE) per g plant powder. 

Total flavonoids were also measured using the aluminum chloride (AlCl_3_) colorimetric assay and were adapted from Zhishen et al. [27]. For this measurement, 100 μL of herbal tea extract were mixed in a 96-well microplate with 6 μL of 5% aqueous sodium nitrite (NaNO_2_) solution. Five minutes later, 6 μL of 10% aqueous AlCl_3_ were added to the preparation before vortex. Following 1 min incubation, forty microliters of 1 M NaOH were added before absorbance measurement at 510 nm. The total flavonoid content was calculated with respect to the (–)-epicatechin (Sigma, Darmstadt, Germany) calibration curve (calibration range 6.25–300 µM) and the results were expressed as mg (–)-epicatechin equivalent (EE) per g plant powder. 

### 2.4. Determination of Antioxidant Capacity of Plant Extracts 

The antioxidant activity of *Antirhea borbonica* plant extract was investigated by using the oxygen radical absorbance capacity (ORAC) and the 2,2-Diphenyl-1-picrylhydrazyl (DPPH) radical scavenging assays. 

The ORAC assay using fluorescein as the fluorescent probe measures the antioxidant capacity of samples to protect fluorescein from 2,2′-azobis [2-methyl-propionamidin] dihydrochloride AAPH-induced oxidative damage. Briefly, 25 µL of polyphenol extract (at different dilution from 1/2 to 1/200 (in phosphate buffer 75 mM, pH 7.4)) were incubated with 150 µL of fluorescein solution (80 nM). The fluorescence kinetic of fluorescein (Sigma, Darmstadt, Germany) was followed for 2 h at 37 °C at an excitation wavelength of 485 nm and an emission wavelength of 530 nm, after adding 25 µL of AAPH (Sigma, Darmstadt, Germany) solution (150 mM) in each well. The results were based on the area under the curve of fluorescence decay over time and compared with the Trolox calibration curve (concentration range: 6–50 μM). Free radical-scavenging activities of polyphenol-rich plant extracts were expressed as mM Trolox (Sigma, Darmstadt, Germany) equivalent.

DPPH protocol is based on that described by Yang et al. with few modifications [28]. Briefly, 200 µL of 0.3 mM methanolic solution of DDPH were incubated in a 96-well microplate with either 40 µL of herbal tea extract (about 12 mM GAE, 40 µL of 10 mM gallic acid (Sigma, Darmstadt, Germany) (dilution in DMSO) used as standard polyphenol or 40 µL of 10 mM ascorbic acid (Sigma, Darmstadt, Germany) (dilution in H_2_O) and 40 µL of 8.5 mM caffeic acid (Sigma, Darmstadt, Germany) as standard antioxidants. After 30 min incubation in the dark at 25 °C, absorbance was measured at 517 nm and free radical-quenching activity of DPPH (percentage) was calculated by using the following formula:

Antioxidant capacity (%) = (A_0_ − A_1_)/A_0_ × 100

where A_0_ is the absorbance of the reaction mixture and A_1_ the absorbance of the reaction mixture in the presence of the plant extract.

### 2.5. BSA and AGE Preparation and Biochemical Characterization

Glycation of bovine serum albumin (Sigma-Aldrich, Darmstadt, Germany) was performed by incubating 15 mL of BSA (20 g/L in PBS) in the presence of 110 µL of methylglyoxal 40% (MGO, final concentration 7.5 mM) and completed with 15 mL phosphate-buffered saline (PBS; 0.2 M, pH 7.4) containing or not *Antirhea borbonica* (*Ab*) plant extract (final concentration 360 µM of GAE). The incubation was performed for BSA (wo. MGO/wo. *Ab*), BSA + *Ab* (wo. MGO/w. *Ab*), BSA + MGO (w. MGO/wo. *Ab*) and BSA + MGO + *Ab* (w. MGO/w. *Ab*) during 7 days under sterile condition (filtration with 0.2 µm Millipore membrane) at 37 °C in the dark and under agitation. All the incubations were performed in triplicates. After incubation, methylglyoxal and plant extract in excess were removed by extensive dialysis against PBS. Protein content of albumin preparations were checked by using bicinchoninic acid assay (BCA) and albumin samples were analyzed by native PAGE (12.5% of acrylamide) and denaturing PAGE (12.5% of acrylamide) and stained with Coomassie blue. Then, samples were stored at –80 °C for further analysis. The antiglycation and antioxidant potential of herbal extract was determined by assessing the level of fructosamines, fluorescent AGE, free amino groups, free sulphydryl groups, tryptophan quenching and β-amyloid aggregation. 

### 2.6. Fructosamine and Fluorescent AGE Determination

Fructosamine content in our preparations was determined according to a protocol previously described by our group [9]. Briefly, the reaction consists of the reduction of nitroblue tetrazolium (NBT) and the consequent change in absorbance is measured at 530 nm. Results were expressed in nanomol of 1-deoxy-1-morpholinofructose (DMF) according to a calibration curve using a synthetic ketoamine as a standard. 

The fluorescence emission intensity of AGE product was obtained with 370 nm excitation wavelength using a spectrophotometer (FluoroMax-4, Horiba, Kyoto, Japan) with excitation and emission slits set at 5 nm [29]. Samples are diluted at 1.5 mg/mL in 50 mM sodium phosphate buffered saline (PBS, pH 7.4). Percentage of AGE formation was determined using the following formula:

AGE (%) = (ImaxG − ImaxG0)/ImaxG0 × 100

where AGE% represents the relative percent of AGE, ImaxG is maximal fluorescence intensity of glycated albumin and ImaxG0 is the maximal fluorescence intensity of nonglycated albumin (BSA).

### 2.7. Free Primary Amino and Sulphydryl Group Determination

Quantification of primary free amino groups in our albumin preparation used 2,4,6-trinitrobenzene sulfonate (TNBS) reagent which forms color when complexing with free amino groups [9]. A calibration curve was established by using increasing concentrations of L-glycine up to 2 mM (Sigma-Aldrich, Darmstadt, Germany). Absorbance was read at 420 nm and results were expressed as mol of amine/mol of protein.

Ellman’s assay containing 5,5-dithiobis(2-nitrobenzoic acid (DTNB) was used to determine thiol group number in albumin preparations. Protocol was well described in a previous study [9]. Briefly and for each assay, a calibration curve was established by using increasing concentrations of L-cysteine up to 100 nmol (Sigma-Aldrich, Darmstadt, Germany). Thiol group numbers for each albumin preparation was determined in duplicate on two different sample dilutions after absorbance measurement at 412 nm. Results were expressed as the number of free -SH group per mol of BSA.

### 2.8. Quenching of Intrinsic and Thioflavin T Fluorescence Determination

The intrinsic fluorescence of albumin is mainly attributed to both tryptophan residues present in BSA molecule. The maximum emission of intrinsic fluorescence was determined for our albumin samples (1.5 mg/mL in PBS) from fluorescent emission spectra obtained with Horiba FluoroMax-4 spectrophotometer (250–500 nm range) under excitation wavelength at 270 nm (slit, 5 nm) [30]. The relative percent of quenching of tryptophan fluorescence was calculated using the following formula:

Trp quenching (%) = (Imax_Trp_ – Imax_Trp0_)/Imax_Trp0_ × 100

where Imax_Trp_ is maximal fluorescence intensity of albumin sample and Imax_Trp0_ is the maximal fluorescence intensity of native non-glycated albumin (BSA).

For β-aggregation determination, thioflavin T (ThT) a specific fluorescent probe for amyloid cross β structure was used [29]. Albumin samples (2.5 µM) were incubated with 30 µM thioflavin T solution (dilution in H_2_O) for 1 h at room temperature. The thioflavin emission spectra were obtained in the range of 250–600 nm under excitation at 435 nm (slit, 5 nm). The relative percent of β-amyloid formation was calculated using the following formula:

β-aggregation n (%) = (Imax_ThT_ − Imax_ThT0_)/Imax_ThT0_ × 100

where Imax_ThT_ is maximal thioflavin T fluorescence intensity of albumin samples and Imax_ThT0_ is the maximal thioflavin fluorescence intensity of nonglycated albumin (BSA).

All fluorescence spectra were corrected for the respective different absorption.

### 2.9. Erythrocyte Preparations

Erythrocytes were obtained from the French blood national agency (EFS-LR agreement number # 2018001378). Concentrated red blood cells were washed 3 times with sterile isotonic solution (NaCl 0.15 M, pH 7) and prepared at 20% hematocrit in phosphate buffered saline solution/5 mM glucose (PBS/0.1% glucose) in the presence or absence of *Ab* plant extract (70 µM GAE) and in the absence or presence of 7.5 mM of methylglyoxal solution. After 24 h of incubation at 37 °C, erythrocytes were washed 3 times with NaCl 0.15 M before a direct use by FACS or ektacytometry. 

### 2.10. Zebrafish Maintenance and Treatment

Adult AB wild type zebrafish (3 to 6 months) were housed in the zebrafish facility of the CYROI/DéTROI (La Réunion) and maintained under standard conditions of temperature (28.5 °C), photoperiod (14 h dark/10 h light), pH (7.4) and conductivity (400 μS). Zebrafish were fed daily with commercially available dry (GEMMA 300, Planktovie). All animal experiments were done in CYROI (UMR 1188) and conducted in accordance with the French and European Community Guidelines for the Use of Animals in Research (86/609/EEC and 2010/63/EU) and approved by the local Ethics Committee for animal experimentation of CYROI and the French Government (APAFIS # 2019110510533837_v5).

Zebrafish were divided into three groups—(1) control fish, (2) MGO-treated fish and (3) MGO-treated fish + *Ab*. Prior to MGO injection, the third group was treated for 48 h with *Antirhea borbonica.* The herbal tea treatment was renewed every 24 h. The two other groups were placed in normal fish water for the 48 h. After 48 h, the first group of fish was injected with 1X PBS (Control fish), the second one with 2.5 µL of MGO (100 mg/kg of body weight) and the third one was injected with 2.5 µL of MGO (100 mg/kg of body weight) and treated one more day with *Ab* in order to investigate the effects of *Antirhea borbonica* on red blood cells glycation (See Figure S1).

Twenty-four hours post-injection, the fish were euthanized using tricaine (MS 222-Sigma). Fish were gently dried with a tissue and one eye was removed allowing the ocular cavity to fill with blood. The blood from 2 fish was collected into 50 µL of 1X PBS containing EDTA in order to avoid blood clotting. For each independent experiment, three pools of blood collected from 2 fish were performed for each condition (Control, MGO, MGO + *Ab*) and three independent experiments were performed.

### 2.11. Mass Spectroscopy for Hemoglobin Glycation Level Determination

Glycation of both α and β hemoglobin subunits was analyzed by matrix-assisted desorption/ionization time-of-flight mass spectrometry (MALDI-TOF MS) for mass shift determination as previously described [31]. Mass spectra were obtained in three independent experiments. On each spectrum, the four main peaks for α and β hemoglobin subunits and their glycated forms were identified. For each peak, the mass (*m/z*) and Δ_mass_ between non-glycated and glycated subunits were obtained. Relative intensity of each peak was calculated as follow—relative % glycation = (intensity glycated-hemoglobin/intensity hemoglobin) × 100.

### 2.12. Erythrocyte Sensitivity to Hemolysis Determination

After 24 h incubation, erythrocyte preparations were washed with NaCl 0.15 M. After centrifugation at 2000× *g* for 10 min, absorbance of supernatant was measured at 450 nm on a microplate reader (FLUOstar Optima, BMG Labtech, Ortenberg, Germany). The percentage of free hemoglobin resulting from lysis of the cells was calculated in percentage to total hemolysis measured on 10% hematocrit erythrocyte preparations incubated with deionized water.

### 2.13. Flow Cytometry Assays

Erythrocyte shape, eryptosis and intracellular reactive oxygen species (ROS) production in our different erythrocyte preparations (from human and zebrafish) were determined by flow cytometry using Beckman Coulter’s CytoFLEX and Cytexpert software (v2.1, Beckman Coulter, Brea, CA, United States). A specific erythrocyte cell population was selected by gating and could be characterized by its typical location in a forward scatter (FSC) versus a side scatter (SSC) parameter graph. For phosphatidylserine exposure determination, 100 µL of erythrocytes (1/50 dilution) were preliminary incubated with 2 μg/mL Annexin V-FITC in binding buffer (BioLegend, San Diego, CA, United States) for 30 min at RT before flow cytometry analysis. Annexin V protein exhibits a high affinity for phosphatidylserine (PS) and was measured with an excitation wavelength of 488 nm and an emission wavelength of 530 nm. For the evaluation of ROS production, 100 µL of erythrocytes (1/50 dilution) were incubated with 2 µM of fluorescent probes, dichlorodihydrofluoresceindiacetate (DCFH-DA; Sigma-Aldrich, Darmstadt, Germany) or dihydroethidium (DHE; Sigma-Aldrich, Darmstadt, Germany) for 30 min at RT.

### 2.14. Shear Stress Gradient Ektacytometry

The determination of erythrocytes membrane deformability was performed using an ektacytometer (LORCCA MaxSis, Mechatronics, Zwaag, The Netherlands), which measures the elongation of red blood cells as a function of an increasing shear stress (elongation curve) or osmotic gradient (Osmoscan curve). 

To obtain elongation curves, red blood cells suspended at 20% hematocrit were diluted 200 times in an iso-osmolar solution of polyvinylpyrrolidone buffer (PVP, viscosity 28.6 mPa/s). Deformation was expressed as an elongation index (EI) calculated for 19 shear-stress intensities between 0.30 and 80 Pa (increasing rotation speed) as follow—EI = (A – B)/(A + B). In this formula, A and B represent the length and the width of the ellipsoid diffraction pattern, respectively. The deformability curve obtained by plotting the calculated values for EI versus the shear-stress (SS) [32] was analyzed using the Lineweaver-Burke model which links the shear-stress and the elongation index according to the following equation:

$$\frac{1}{\mathrm{EI}}=\frac{{\mathrm{SS}}_{1/2}}{{\mathrm{EI}}_{\max}}\times \frac{1}{\mathrm{SS}}+\frac{1}{{\mathrm{EI}}_{\max}}$$

From this equation, the maximum elongation index (EI_max_) and the shear stress at half maximal deformation (SS_1/2_) can be calculated. Both parameters appeared to be relevant indicators of erythrocyte deformability capacity [32].

### 2.15. Osmotic Gradient Ektacytometry

For the acquisition of the osmoscan curve, 200 µL of red blood cells suspended at 20% hematocrit were suspended in 5 mL PVP buffer. A constant shear stress (30 Pa) was applied during the EI measurement with a gradual increase in osmolality from 80 to 500 mOsmol/kg. The resulting osmotic gradient curves reflect red blood cells deformability as a continuous function of suspending medium osmolality. Different parameters obtained from these curves were analyzed, such as the minimal elongation index value measured at low-osmotic environment (Ei_os-min_) and maximum elongation index values corresponding to the maximal deformability obtained near the isotonic osmolality (Ei_os-max_). From these parameters, the ratio of maximal and minimal EI values was calculated—rEI = Ei_os-max_/Ei_os-min_. This ratio value can be interpreted as the amplitude of deformability of erythrocytes [33].

### 2.16. Statistical Analysis

Data were expressed as the mean ± standard error of the mean (SEM) from at least three independent experiments performed in triplicate. Statistical analyses were performed with Prism (GraphPad Software Inc., San Diego, CA, USA). Statistical significance was determined using one-way ANOVA followed by Dunnett’s test, with a *p*-value < 0.05 required for significance.

## 3. Results

### 3.1. Phenolic Composition of Antirhea borbonica Plant Extracts

*Antirhea borbonica* (*Ab*) medicinal plant is traditionally used in infusion in order to reduce the incidence of obesity and diabetes in the Indian Ocean area. The identification of polyphenols of aqueous extract of *Ab*, obtained by infusion of the plant, was determined by mass spectroscopy. Figure S2 shows the total ion chromatogram (TIC) and chromatographic profiles of this extract recorded at 280 nm and 320 nm. A total of 12 main compounds, numbered according to their elution order, were identified and reported in Table 1 with their retention time, experimental *m/z* mass and corresponding chemical formula. The main identified polyphenols were two caffeic acid derivatives (chlorogenic and dicaffeoylquinic acids) and several minority polyphenols were also identified, such as flavonoids (quercetin, kaempferol and quercetin derivatives) and hydroxybenzoic acid (gallic acid and protocatechuic acid).

Total polyphenol, flavonoid contents and antioxidant capacity of Ab were also evaluated. As mentioned in Table 2, *Ab* aqueous extract is characterized by high phenolic (7.69 ± 0.59 mg GAE/g) and flavonoid (2.70 ± 0.04 mg EE/g) contents associated with an antioxidant capacity of 16.30 ± 2.73 mM Trolox equivalent (ORAC assay). DPPH assay was also performed to determine whether polyphenol presence was associated with antioxidant properties of the extract. Data confirmed that the *Ab* extracts exert a high free radical scavenging activity reaching 82.6 ± 2.71% of inhibition (Table 3) which is quite equivalent to ROS scavenging activity exerted by 10 mM of ascorbic acid (93.2 ± 3.30%) or by 10 mM of gallic acid (92.0 ± 2.54%) and higher than 8.5 mM of caffeic acid ROS scavenging activity (41.22 ± 1.22%).

### 3.2. Antirhea borbonica Plant Extracts Prevent Albumin from Glycation and Oxidation

Electrophoretic analysis showed that albumin samples exhibited approximately identical molecular weights (about 66 kDa), with a slight variation of a few daltons for MGO-modified BSA (cf SDS PAGE results in Figure S3A). By contrast, BSA samples exhibited different net charges, as confirmed by native polyacrylamide gel electrophoresis (cf native PAGE results in Figure S3B). A higher migration was observed for MGO-modified BSA samples compared to unmodified BSA. This enhanced migration, reflecting impairment in albumin isoelectric point, was reduced when glycation was performed in the presence of *Ab*.

The effect of *Ab* aqueous extracts was investigated on multiple parameters reflecting albumin glycation or oxidation. AGE, free amine, ketoamine levels and free thiol group contents in albumin are reported in Table 4. As expected, an incubation with 7.5 mM of methylglyoxal for 7 days induced a marked increase in ketoamine levels (6.30 ± 0.44 mol/mol vs. 0.07 ± 0.29 mol/mol for BSA, *p* < 0.001) and in fluorescent AGE levels (+708 ± 193%, *p* < 0.001) compared to native BSA. These increases were associated with a significant drop in thiol group (0.384 ± 0.024 mol/mol vs. 0.968 ± 0.089 mol/mol for BSA, *p* < 0.01) and free amine group contents (46.9 ± 1.06 mol/mol vs. 58.9 ± 2.43 mol/mol for BSA, *p* < 0.01) reflecting thiol oxidation and primary amino group glycation. 

In the presence of *Ab* aqueous extract (615 µM of GAE), early (ketoamine) and terminal (AGE) glycation product levels in MGO-induced glycated BSA (BSA + MGO) were found to be significantly reduced. Indeed, the plant extract induced a significant reduction in ketoamine (–37%, *p* < 0.05) and fluorescent AGE (–53%, *p* < 0.05) levels. Similarly, *Ab* protects free amino groups from glycation. Indeed, AGE-albumin displayed ~46.9 free amino group and this level rose significantly (*p* < 0.05) to ~49.5 in the presence of the plant extract (BSA + MGO + *Ab*). The impact of plant extract on free thiol protection was limited with an increase of ~+11% that did not reach statistical significance. Taken together, these results show that *Ab* exert antiglycative properties for albumin.

Of note, if the sole *Ab* plant extract did not have significant influence on ketoamine, fluorescent and free amino group levels on native BSA (BSA + *Ab*), it decreased drastically the thiol protection capacity of the protein (0.178 ± 0.023 mol/mol vs. 0.968 ± 0.089 mol/mol, *p* < 0.001).

### 3.3. Antirhea borbonica Plant Extracts Impact on Albumin β-Aggregation

As reported in previous studies, albumin glycation using methylglyoxal as a glycative molecule was associated with the formation of very stable high molecular mass aggregates with high level of amyloid cross β-structure [8]. The capacity of the aqueous plant extract to prevent albumin from β-amyloid-type aggregation during glycation was investigated by using Thioflavin (ThT) marker. The measurement of maximum fluorescence emission (between 487 and 500 nm) obtained from ThT fluorescence spectra (Figure S4A) and performed to detect specifically β-fibrillar structure in albumin featured a significant increase for BSA + MGO (+722% vs. BSA, *p* < 0.001) but also for BSA in the presence of *Antirhea borbonica* (BSA + *Ab*) (+1725% vs. BSA, *p* < 0.001) (Table 4). By contrast, the plant extract did not significantly further increase β-amyloid formation observed for BSA + MGO (+849 ± 198% vs. +722 ± 64.9%, *p* = 0.48). The potent stimulation of *Ab* in β-aggregation could be explained by the involvement of the plant in albumin tertiary conformational changes as observed with the intrinsic fluorescence measurements. The fluorescence spectra featured in Figure S4B, show typical band at about 350 nm mainly attributed to both tryptophan residues (Trp-134 and Trp-212). Tyrosine residues of the protein contribute also to this fluorescence. But, at 270 nm excitation wavelength, tyrosine residues fluorescence remains weak. In comparison with BSA fluorescence spectra, all modified albumins undergo differential quenching of intrinsic fluorescence as a consequence of protein incubation with MGO, *Ab* or both. The different percentages of fluorescence quenching reported in Table 4 reflect the conformational alteration of albumin associated with β-structure aggregate formation. 

### 3.4. Effects of Antirhea borbonica Plant Extracts on MGO-Induced Hemoglobin Glycation

To determine the glycation level in erythrocytes samples, relative glycation percentage of the alpha and beta forms hemoglobin were investigated by mass spectroscopy. Representative mass spectra of alpha and beta-hemoglobin subunits and their glycated forms are featured in Figure S5. Relative percentage of glycation for both hemoglobin subunits calculated from relative intensity of peaks of non-glycated and glycated forms are presented in Table 5. Of note, percentages of glycated hemoglobin reported in this table using mass spectrometry (between 18% and 25% for untreated red blood cells) cannot be compared to HbA1c range values obtained in clinical test. Here relative glycation percentage results from the ratio between the peak intensity obtained in the mass spectra for the glycated isoform and the non-glycated isoform as described in the Method section. The incubation with 7.5 mM of MGO induced a marked increase in hemoglobin glycation for the alpha (25.0 ± 1.8% vs. 18.0 ± 0.9% for RBC, *p* < 0.05) and beta (33.0 ± 2.9% vs. 25.0 ± 1.1% for RBC, *p* < 0.05) subunits. However, no significant effect in hemoglobin glycation is observed when erythrocytes were co-exposed to *Ab* extracts and MGO.

### 3.5. Antirhea borbonica Plant Extracts Prevent Erythrocytes from MGO-Induced Injuries 

Because of its very high reactivity, methylglyoxal is known as a potent source of ROS that could lead to detrimental effects on erythrocytes such as hemolysis or eryptosis. During experimental conditions set up, an increasing range of MGO concentrations (0 to 10 mM) was tested on erythrocyte properties in term of deformability capacity, eryptosis and oxidative stress. Results displayed on Figures S6 and S7A,B show a progressive increase in the impact of MGO on erythrocyte properties which becomes significant when 7.5 mM MGO concentration was used.

**Figure 1:**
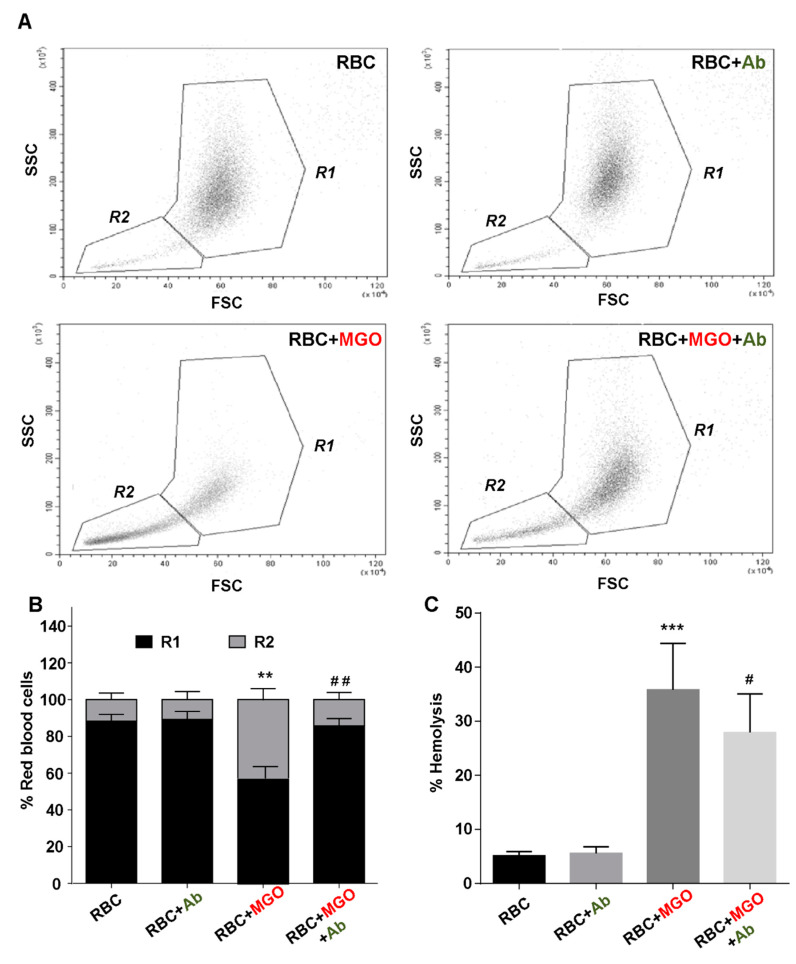
Protective effect of *Antirhea borbonica* on erythrocytes morphology. Red blood cells morphology was investigated by flow cytometry. (**A**) Erythrocyte populations were gated according to cell location in a forward scatter (FSC) versus a side scatter (SSC) parameter. R1 and R2 gates represented respectively mature and senescent/aged erythrocytes for RBC (red blood cells); RBC + *Ab* (in green color); RBC + methylglyoxal (MGO, in red color) and RBC + MGO + *Ab*. (**B**) Average percentage of mature erythrocytes (R1) and altered erythrocytes (R2). (**C**) Average percentage of hemolysis induced by the change in solution tonicity (PBS→NaCl 0.9%). Data are mean ± SEM of five independent experiments. *Effect of MGO (vs. RBC), *** *p* < 0.001, ** *p* < 0.01. ^#^ Effect of *Ab* (vs. RBC + MGO), ^##^ *p* < 0.01, ^#^ *p* < 0.05.

To assess the effects of *Ab* on methylglyoxal-induced toxicity to erythrocytes, the morphology red blood cells incubated 24 h in the presence or absence of MGO and/or *Ab* extract were analyzed by FACS. Both gates (R1 and R2) set on the FSC vs. SSC dot plots featured in Figure 1A, allowed to distinguish two erythrocytes populations corresponding to mature/intact erythrocytes (R1) and senescent/altered erythrocytes exhibiting rather low FSC and SSC values (R2). The percentage of the two respective populations is given in Figure 1B for each erythrocyte condition. As hypothesized, incubation with methylglyoxal caused a significant increase in the senescent erythrocyte population (42.8 ± 6.35% vs. 11.7 ± 3.63% for RBC, *p* < 0.01). The presence of *Ab* significantly reduces the percentage of this altered erythrocyte population up to 14.3% (*p* < 0.01). 

As illustrated in Figure 1C, methylglyoxal also exerts a damaging effect on erythrocytes by inducing a significant increase in cell hemolysis (35.8 ± 8.58% vs. 0.80 ± 3.63% for RBC, *p* < 0.001). Then and in the presence of the plant extract, the observed hemolysis was found to be partially reduced (up to 26.90 ± 7.14%, *p* < 0.05). Noteworthy enough, in both results, the sole *Ab* extract did not exert any significant detrimental effect on red blood cells attesting the absence of any side effect of the plant in our experimental conditions. 

### 3.6. Antirhea borbonica Plant Extracts Preserve the Deformability Capacity of Erythrocyte Membrane from Glycation Impairments 

The changes in erythrocyte morphology and membrane integrity were investigated by ektacytometry. The membrane deformability was assessed according to two criteria—its resistance to enhanced shear stress intensity and to increasing osmolarity gradient.

**Figure 2:**
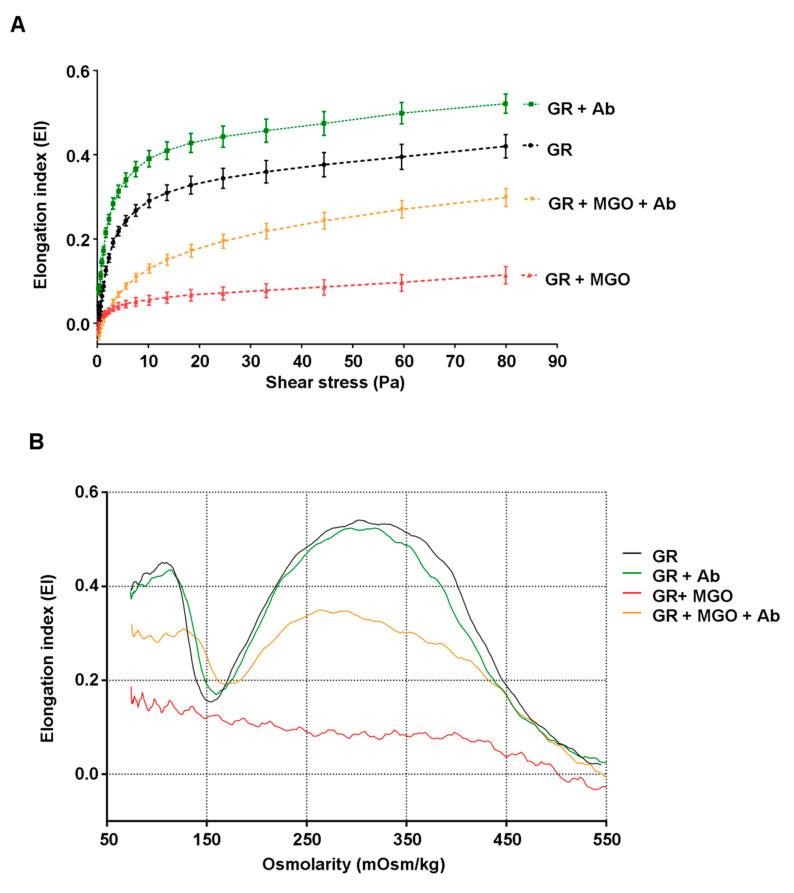
*Antirhea borbonica* prevents erythrocytes from deformability impairment induced by MGO. (**A**) Curves represent erythrocyte deformation as elongation index as a function of shear stress for RBC (black line); RBC + *Ab* (green line); RBC + MGO (red line) and RBC + MGO + *Ab* (orange line) erythrocyte samples. Data are means ± SEM of six independent experiments. (**B**) Representative osmoscan profiles of RBC (black line); RBC + *Ab* (green line); RBC + MGO (red line) and RBC + MGO + *Ab* (orange line) erythrocyte samples.

Figure 2A showed typical curves corresponding to elongation index values (EI) as a function of increasing shear stress intensity in isotonic condition. Table 6 reports two pertinent elongation parameters (Ei_max_: EI at infinite shear stress and SS_1/2_: half-maximal deformation) that reflect the properties of the entire curve and enable an efficient comparison between different erythrocyte preparations. Normal RBC exhibit characteristic elongation curves of normal cell deformation with increasing stress tending towards an ellipsoidal shape. Overall, MGO-modified red blood cells display lower EI values than normal RBC and for all levels of applied shear stress. We can also note that, in the presence of MGO, the more glycoxidant used during incubation with RBC, the more the loss in deformability (Figure S6). The calculation of standardized deformability parameters gives for RBC + MGO a lower Ei_max_ value (0.113 ± 0.022) and higher SS1/2 value (12.3 ± 4.28) than for normal RBC (Ei_max_: 0.428 ± 0.038 and SS_1/2_: 4.45 ± 0.978). Thus, these two indicators reflect the loss in the capacity of RBC + MGO to be optimally deformed. In the presence of *Antirhea borbonica* plant extract, altered erythrocytes exhibit an elongation curve with higher EI values and therefore lead to marked increase of Ei_max_ value (0.239 ± 0.103, *p* < 0.01). These last data reflect that in these conditions, red blood cells are prone to a better deformability capacity. 

To support the statements made with previous results, osmotic gradient ektacytometry was also performed on erythrocyte preparations. With this method, elongation index was measured at a constant shear stress and under a gradual increase in osmolality, allowing the acquisition of characteristic osmoscan curves (Figure 2B).

The maximum (Ei_os-max_) and minimum (Ei_os-min_) points represent the maximal and minimal deformability measures at low-osmotic and isotonic environment, respectively. Both quantitative parameters and the calculated ratio (rEI = Ei_os-max_/Ei_os-min_) are given in Table 6 and could be considered as pertinent indicators of RBC deformability capacity in variable osmotic condition. For normal RBC, the osmoscan curve displayed a minimum EI (0.200 ± 0.064) at about 150 mOsm/kg and a maximum of deformability (0.494 ± 0.066) near 300 mOsm/kg at isosmolar condition giving a ratio value (rEI) around 2.5. In the presence of the sole *Ab* extract, erythrocytes display a quite similar pattern with the same values for quantitative parameters. By contrast, incubation with MGO produced an extremely irregular curve that is almost not evaluable, allowing only an EI_os-max_ value around 0.206 to be determined. Interestingly, in the presence of *Ab* extracts, the osmoscan curve of MGO-induced erythrocytes becomes regular again and all quantitative parameters could be determined. For instance, the EI_os-max_ value was found to be significantly higher than for altered red blood cells (0.302 ± 0.032 vs. 0.206 ± 0.035, *p* < 0.05) and the rEI reached the value of 1.58 showing that with the plant extract, erythrocytes partially recover their deformability capacity.

### 3.7. Antirhea borbonica Plant Extracts Protects Erythrocytes from MGO-Induced Oxidative Stress and PS Exposure

**Figure 3:**
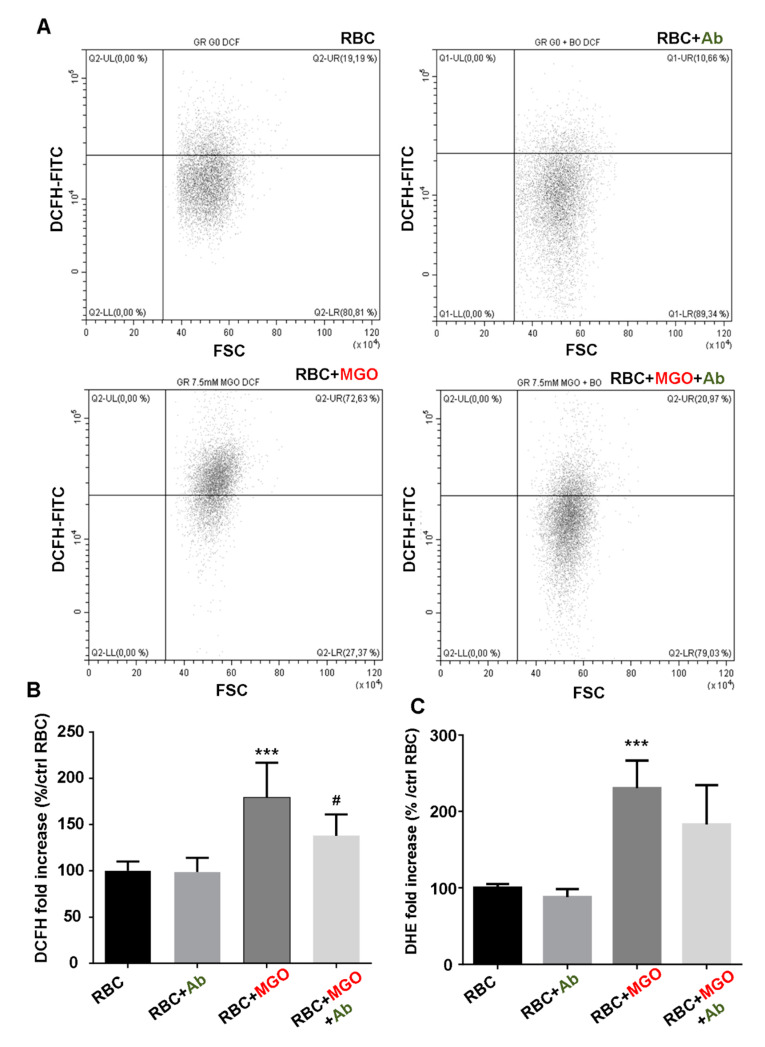
Antioxidant effect of *Antirhea borbonica* on MGO-induced erythrocyte glycation. Intracellular ROS levels in mature erythrocytes were measured by flow cytometry using DCFH-DA and DHE fluorescent probes. (**A**) Typical FACS dot plots after DCFH staining of RBC; RBC + *Ab* (in green color); RBC + MGO (in red color) and RBC + MGO + *Ab* erythrocytes. (**B**) Average fold increase in DCFH fluorescence compared with RBC. Data are mean ± SEM of six independent experiments. * Effect of MGO (vs. RBC), *** *p* < 0.001. ^#^ Effect of *Ab* (vs. RBC + MGO), ^#^ *p* < 0.05. (**C**) Average fold increase in DCF fluorescence compared with RBC. Data are mean ± SEM of four independent experiments. * Effect of MGO (vs. RBC), ^***^ *p* < 0.001.

Enhanced cell hemolysis and membrane deformability impairments may be due to enhanced oxidative stress in MGO-glycated erythrocytes. To test this hypothesis, intracellular ROS level, leading to oxidative stress, was investigated by FACS using two fluorescent probes—DCFH-DA and DHE. The dot plot (DCFH-FITC vs. FSC) in Figure 3A showed a distinct erythrocyte population which globally exhibited a higher DCFH fluorescence in the presence of MGO than normal RBC or RBC incubated with *Ab* extracts. This significant MGO-induced ROS level increase is showed with both DCFH (+79.2 ± 37.4% vs. RBC, *p* < 0.001) and DHE (+130.5 ± 36.2% vs. RBC, *p* < 0.001) probes (Figure 3A,B, respectively). It should be noted that incubation with increasing concentrations of methylglyoxal induced a dose-dependent elevation in DCFH fluorescence reflecting elevating intracellular ROS levels (Figure S7A). 

**Figure 4:**
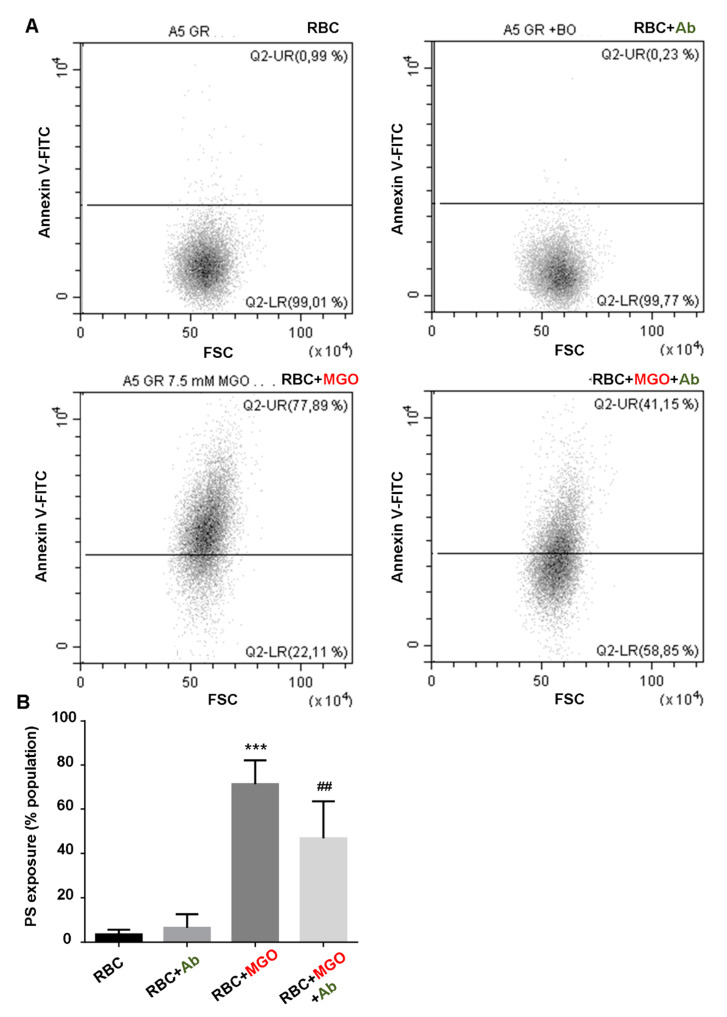
*Antirhea borbonica* protects erythrocytes from MGO-induced eryptosis. Phosphatidylserine exposure (PS) was investigated by flow cytometry by using annexin V-FITC fluorescent probes. (**A**) Typical representative FACS dot plots after annexin V staining of RBC; RBC + *Ab*; RBC + MGO and RBC + MGO + *Ab* erythrocytes. (**B**) Average percentage of PS exposure compared with RBC. Data are mean ± SEM of six independent experiments. * Effect of MGO (vs. RBC), *** *p* < 0.001. ^#^ Effect of *Ab* (vs. RBC + MGO), ^##^ *p* < 0.01.

The capacity of *Ab* plant extract to reduce significantly the intracellular oxidative stress induced by MGO was observed with DCFH probe (Figure 3A, low right panel). Indeed, in these conditions, the increase of DCFH fluorescence was roughly halved in comparison with altered RBC (+37.9 ± 22.9% vs. +79.2 ± 37.4% for RBC + MGO, *p* < 0.05) (Figure 3B). These results confirm the antioxidant potential of the plant. Phosphatidylserine (PS) exposure at erythrocyte surface reflects eryptosis state. PS exposure was considered as a key “eat signal” for erythrocyte clearance by phagocytes [34]. The intracellular oxidative stress previously evidenced in our erythrocyte preparations could be a determinant eryptosis inducer. We checked this hypothesis by investigating the percentage of FITC-annexin V-positive erythrocytes using flow cytometry. Corresponding dot plots (annexin V-FITC vs. FSC) and average percentage of positive PS exposure population are reported in Figure 4. Incubation with methylglyoxal increased significantly and in a dose-dependent manner, the PS exposure (Figure S7B). With 7.5 mM of MGO this increase reached more than 70% of erythrocyte population (71.35 ± 10.76% vs. RBC, *p* < 0.001). This triggered eryptosis by MGO treatment was significantly reduced with *Ab* plant extract as far as the PS positive erythrocytes percentage was reduced until 46% (46.78 ± 12.76% vs. RBC + MGO, *p* < 0.05).

### 3.8. In Vivo Effects of Antirhea borbonica in Zebrafish 

**Figure 5:**
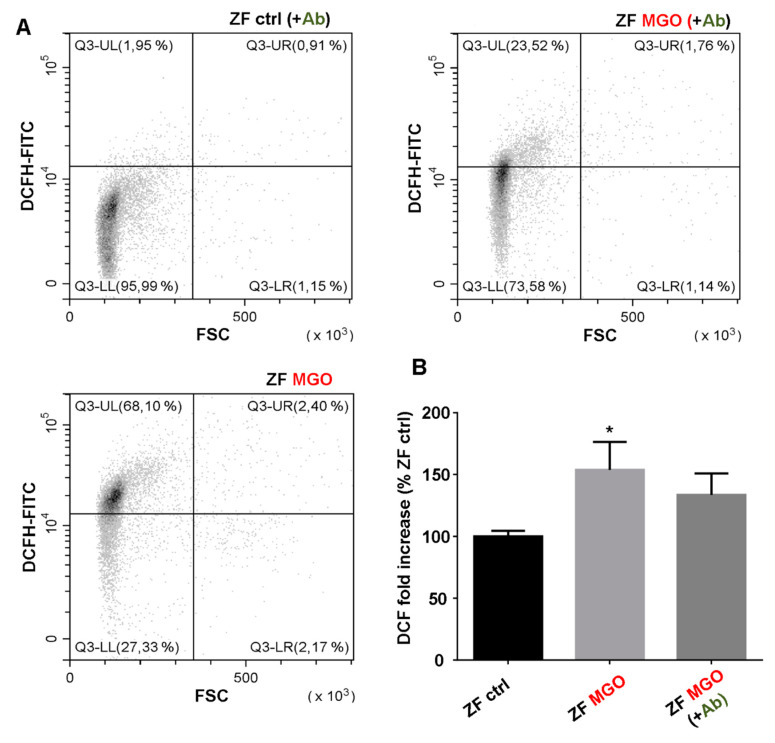
Antioxidant effect of *Antirhea borbonica* on red blood cells of MGO-induced oxidative stress in zebrafish. Intracellular ROS levels in zebrafish erythrocytes were investigated by flow cytometry using DCFH-DA probe. (**A**) Typical FACS dot plots FSC vs. DCFH-FITC after red blood cells staining of zebrafish (ZF) ctrl, ZF MGO and ZF MGO + *Ab*. (**B**) Average fold increase in DCFH fluorescence compared with RBC of control. Data are means ± SEM of three independent experiments. * Effect of MGO (vs. ZF ctrl), * *p* < 0.05.

In order to confirm the potential effects of *Ab* on blood glycation in vivo, we took benefit from zebrafish model developed in our laboratory. Indeed, zebrafish share a high genomic homology with humans (>70%) as well as evolutionary conserved physiological processes [35]. In addition, it has been successfully used for studying metabolic disorders such as hyperglycemia and diabetes [36,37]. As shown in Figure 5, the injection of MGO led to a significant increase in red blood cell oxidative stress as revealed by the DCF probes 24 h post-injection. These results were confirmed in 3 independent experiments. In order to substantiate the previous results obtained in vitro for *Ab*, fish were treated for 48 h with *Ab* herbal tea and injected with MGO after this period. The fish were then placed back into a freshly prepared herbal tea for 24 h more before being sacrificed and processed for analyses. As shown in Figure 5, *Ab* exerts potent antioxidant activities as MGO-induced oxidative stress is no more observed in fish that were previously treated with *Antirhea borbonica* extract. 

## 4. Discussion

Glycoxidation is a physiological complex process which is amplified in hyperglycemic conditions. The glycation reaction corresponds to the initial precursor of this process to other even more harmful phenomena such as oxidative stress, inflammation and platelet aggregation. The AGEs accumulation, resulting from this critical process, is involved in progression of diabetes complication such as vascular complications [38].

Albumin and erythrocytes represent abundant circulating components which render them very sensitive to glycoxidative modifications [6]. Albumin and erythrocytes in their native form exert many beneficial properties but when glycoxidized they become deleterious actors that were involved in the development of vascular complications. Indeed, serum albumin, primary glycoxidation target, constitutes an important provider of circulating AGEs and therefore of increased plasmatic oxidative stress. The resulting oxidative damage thus affects the main molecular and cellular actors of atherogenesis, such low-density-lipoprotein (LDL), the main contributor of atheroma plaque development and the weakened endothelium that will facilitate macrophage and lipid infiltration [39,40]. Erythrocyte is now considered as a new culprit contributing to the pathogenesis of atherosclerosis by directly participating in intraplaque hemorrhage and/or thrombus formation [41]. Consequently, inhibition of glycation, oxidation and glycoxidation appears to be the basis of antiatherogenic and antithrombotic strategies to be considered and the discovery and investigation of AGEs inhibitors would offer a potential therapeutic approach for prevention of diabetic complications. Some potential synthetic AGE inhibitors and antiglycant agents were discovered and tested such as metformin or aminoguanidine [42]. But, because of potential toxic and side effects of these synthetic molecules, antioxidant compounds from natural products such as polyphenols were preferred as safe and promising agents for their antiglycation activities and for prevention of AGEs formation.

It appeared that our *Ab* aqueous plant extract exhibited a higher polyphenols and flavonoids content (5.2% GAE (*w/w*) /2.1% EE (*w/w*)) than the acetonic extract of the same plant investigated by Marimoutou et al. [25]. In addition, major phenolic acids including caffeic acids derivatives and flavonoids like quercetin derivatives known to be abundant in medicinal plants were identified in our plant extract [43]. The two major compounds of this extract are caffeic acids derivatives—dicaffeoylquinic acid and chlorogenic acid. The antioxidant activity and ROS trapping capacity of dicaffeoylquinic acid has been reported in the treatment of oxidative damage induced by ROS such as age-related diseases and certain degenerative diseases [44].

Chlorogenic acid delays the intestinal absorption of glucose and therefore its passage through the bloodstream [45]. This phenolic acid also protects LDL from oxidation and thus limits the formation of atheroma plaque, a major vascular complication associated with diabetes [46].

This high polyphenol level could explain the strong free radical-scavenging activity of the plant extracts shown with DPPH and ORAC assays. Indeed, polyphenols were identified as down regulators of ROS production and pro-inflammatory cytokines from H_2_O_2_ exposed preadipocyte cells and LPS exposed adipocyte [24,25]. This beneficial extract prepared in the form of herbal tea and used as therapeutic human diet can also act on the main circulating cells and plasmatic protein. Indeed, many studies showed that several polyphenols including caffeic acid or quercetin derivatives can be absorbed, metabolized after a specific human diet and be detected in plasma the first few hours after ingestion [47,48]. 

In our study, aqueous extract of *Ab* significantly reduced albumin glycation at several stages of this deleterious process. Indeed, the inhibitory effects of this extract acts in the early glycation products (ketoamines) and also in the advanced products (AGEs) formation. Effect of *Antirhea borbonica* was not limited to glycation process inhibition. Indeed, β-structure aggregates formation was found not only with MGO-glycation but also with *Ab* extract. This conformational change of albumin into β-sheet structure induced by the plant suggests a direct impact of *Ab* on the tertiary structure of the protein. This hypothesis is reinforced by the results of tryptophan fluorescence showing an important tryptophan fluorescence quenching of albumin in the presence of the plant. As attested by tryptophan fluorescence quenching phenomenon, a partial unfolding occurs in the presence of MGO or *Antirhea borbonica*, similarly to what was already observed in case of aggregation process of native BSA [49]. In our experimental conditions, this change of tertiary structure could be associated with increasing exposure of hydrophobic pockets usually detected with ANSA fluorescent probe [49]. This increased accessibility of the hydrophobic regions induced by the α-dicarbonyl agent or the plant appears to be favorable for the aggregation process.

These last results showing the direct impact of the rich polyphenol plant extract on albumin structure are consistent with numerous studies showing the affinity of certain polyphenols for albumin, particularly dicaffeoylquinic acid and chlorogenic acid [50,51]. Indeed, numerous polyphenols including phenolic acids or flavonoids are known to exhibit high affinities to serum albumin [52]. In the present study, caffeic acid induced same effects on albumin as *Antirhea borbonica* in terms of tryptophan fluorescence quenching and β-structure aggregates formation (Figure S8). These latest data raise the question of the potential impact of Ab herbal tea on the consequences of such an increase in albumin aggregation.

Several hypotheses can be put forward to explain the inhibition action of *Ab* extract on MGO-induced glycation of albumin. First, polyphenols could directly react with methylglyoxal and then neutralizing its glycating and oxidizing capacity. Indeed, many phenolic acids were found to be able to bind and reduce methylglyoxal molecules [53]. Second, polyphenols and the glycative agent may compete for the sensitive sites of the protein. Indeed, a very recent study by Tagliazucchi et al. showed that some phenolic acids could effectively bind albumin at key glycation sites [54]. 

It is clear that glycation impairs the protective antioxidant properties of albumin [55]. By limiting glycation and thus AGEs formation, the *Ab* extract may protect albumin antioxidant properties. It would be interesting to test the protective effect of *Ab* extract against glycated albumin mediated toxicity to erythrocytes [56].

The observed beneficial effects of *Antirhea borbonica* extract to protect albumin from glycation are comforted by the results highlighting its capacity to prevent red blood cells from oxidative injury. Indeed, MGO-induced oxidative stress damages in erythrocytes represent a determinant starting point that initiate membrane fragility and eryptotic process of red blood cells. This membrane fragility results in increased oxidative mediated hemolysis induced by the α-dicarbonyl agent. We evidenced an effective protection of *Ab* for reducing ROS production, preserving membrane integrity and function and slowing down the senescence process. An enzymatic defense system in erythrocytes could be involved in the protective effect of *Ab*. Indeed, alteration of this protective mechanism in ROS-damaged erythrocytes were already evidenced in H_2_O_2_ or glucose-exposed red blood cells [57]. In addition, several studies report that polyphenols, such as Silymarin and polyphenol-rich extracts from *Achillea* species, could significantly stimulate activities of the antioxidant enzymatic system such as catalase or peroxidase [58,59]. Thus, the return to redox homeostasis with phenolic compounds contributes to reduce lipid peroxidation and preserve membrane integrity [60].

Not only *Ab* extract prevent erythrocytes from oxidative stress and eryptosis and also protect their membrane integrity and hence preserving erythrocyte morphology and deformability capacity. Deformation capacity constitutes a determinant rheological parameter for erythrocytes, which have to pass through tiny capillaries. Then, a relative rigidity of the RBC membrane may provoke significant disruption in the microcirculation efficiency and thus represents a putative contributor to microvasculature occlusion [61].

Here, and for the first time, methylglyoxal as a glycoxidizing agent, was shown to induce severe erythrocyte membrane stiffness in terms of cell deformability capacity following a change in shear stress and also when osmolarity deviates from physiological conditions. In our experimental conditions, if MGO-glycated erythrocytes totally lost their ability to be deformed, *Ab* extract appears to be able to significantly preserve this capacity. RBC membrane disorders causing this loss of deformability may be due to the alteration of specific cytoskeleton proteins such spectrin, ankyrin or protein band.3 [62]. These proteins ensure cohesion between the membrane lipid bilayer and cytoskeleton, thus providing structural and functional integrity of RBC. In oxidative stress situation induced by H_2_O_2_ or AAPH, α- and β-spectrin and band.3 proteins in RBC were shown to be particularly prone to oxidative damages [63,64]. Paiva-Martins et al. showed that many olive oil polyphenolic compounds had the capacity to protect RBC from oxidative injury and suggested that this protection could be mediated by the interaction of 3,4-DHPEA-EDA and hydroxytyrosol with RBC membrane proteins, improving the stability of erythrocytes [64,65]. In our study, methylglyoxal may primarily affect the key proteins, ankyrin, spectrin and band.3, tightly responsible for erythrocyte flexibility and stability. 

The direct consequence of membrane flexibility protection exerted by the *Ab* extract is an increased hemolysis resistance and an improvement in the deleterious morphological change of RBC. Although *Ab* extracts exert potent cytoprotective effects, it does not seem to impact hemoglobin, the main erythrocyte protein. Indeed, by mass spectrometry, we can observe a significant increase in hemoglobin glycation in the presence of methylglyoxal suggesting that the α-dicarbonyl reagent can easily penetrate the membrane and also cause intracellular damage. However, in our experimental conditions, *Ab* extract does not seem to be able to counteract the glycoxidant action of MGO. This conversely action exerted by *Antirhea borbonica* on the membrane versus the intracellular level supports the hypothesis raised above concerning the absence of a direct neutralizing action of the plant on the glycoxidizing agent. The results obtained on the zebrafish model treated with *Antirhea borbonica* support this hypothesis. According to several studies, the hyperglycemia model of zebrafish treated with methylglyoxal appears to be attractive for understanding underlying mechanisms associated with diabetic complications such as microvascular complications [66]. Until now, methylglyoxal in zebrafish was rather tested in the embryonic or larval stage than in adult fish [67]. In our study, a 24 h treatment with MGO through intraperitoneal injection was sufficient to cause intracellular oxidative stress in zebrafish erythrocytes without inducing eryptosis. Furthermore, a 48 h preliminary bathing treatment with *Ab* seems to protect red blood cells against MGO induced oxidative stress. These preliminary results on zebrafish blood seem very promising and further ongoing projects are highly warranted and under progress to decipher the beneficial effect of *Antirhea borbonica* on a diet induced obese zebrafish model. In summary, in the present work, we showed not only that *Antirhea borbonica* extract preserve in vitro erythrocyte from MGO-induced damages and also acts as an in vivo protecting agent in a zebrafish model recently developed in our group [36,68]. If these results cannot be extrapolated to what could be encountered in pathological conditions, several points may in some way relate our in vitro experimental conditions to in vivo situations. Actually, if albumin and erythrocyte glycation was performed by using supraphysiological concentration of the glycating agent, such concentration was chosen to operate a rapid glycation model for long half-life proteins, 21 days and 120 days for albumin and hemoglobin, respectively. These vitro glycation models appeared relevant to what could be encountered in vivo as most of oxidative damages, identified in our in vitro glycated albumin and erythrocytes preparations, were retrieved in albumin and erythrocytes isolated from diabetics [10,15,69].

In addition, the protective effect of Ab was observed in vivo in zebrafish which water bath comprised 0.5 g of plant extract in 2 L. In term of comparison, when used as an herbal tea, 4 g of the plant tea leaves are used for infusion into 1 L [23].

## 5. Conclusions

Aqueous extract of *Antirhea borbonica,* rich in caffeic acid and quercetin derivatives, confer potent antiglycative and antioxidant protection for albumin and erythrocytes. Antiglycative action on albumin was associated with intrinsic conformational changes in favor of aggregative process of the protein. Also, *Ab* extract preserved erythrocyte flexibility and resistance toward a glycative stress attack. These results reveal antioxidant and antiglycant properties of *Ab* that could be used for the dietary modulation of oxidative stress and glycation in hyperglycemic situations.

## Figures and Tables

**Table 1:** Polyphenols identified in *Antirhea borbonica* plant extract.

Peak N	Compound Name	Retention Time (min)	[M – H]	Formula
1	Gallic acid (traces)	1.2	169.0142	C_7_H_5_O_5_
2	Chlorogenic acid	2.7	353.0878	C_16_H_16_O_9_
3	Chlorogenic acid (isomer)	3.5	353.0878	C_16_H_16_O_9_
4	Quercetin hexoside	5.0	463.0882	C_21_H1_9_O_12_
5	Kaempferol hexoside	5.0	593.1512	C_21_H_19_O_11_
6	Kaempferol hexoside (isomer)	5.2	593.1512	C_21_H_19_O_11_
7	Kaempferol hexoside (isomer)	5.4	593.1512	C_21_H_19_O_11_
8	Dicaffeoylquinic acid	6.1	515.1195	C_25_H_23_O_12_
9	Dicaffeoylquinic acid (isomer)	6.4	515.1195	C_25_H_23_O_12_
10	Chlorogenic acid (isomer)	6.6	353.0878	C_16_H_16_O_9_
11	Dicaffeoylquinic acid (isomer)	6.6	515.1195	C_25_H_23_O_12_
12	Quercetin	7.4	301.0354	C_15_H_9_O_7_

Polyphenol-rich plant extract was analyzed by using a Q Exactive Plus mass spectrometer. Compounds were identified according to their retention time (min)/molecular weight (Da) (see spectra on Figure S1).

**Table 2:** Total polyphenol, flavonoid content and antioxidant activity of *Antirhea borbonica* plants extracts.

	Total Polyphenol Content (mg GAE/g Plant)	Total Flavonoid Content (mg EE/g Plant)	Radical Scavenging Capacity (mM Trolox eq.)
*Antirhea borbonica* plant extract	7.69 ± 0.59	2.70 ± 0.04	16.30 ± 2.73

Phenolic acids and flavonoids levels were determined by using colorimetric assays and expressed as mg gallic acid equivalent (GAE)/g plant dry powder or mg epicatechin equivalent (EE)/g plant dry powder, respectively. Free radical-scavenging activities was assessed by ORAC assay and expressed as mM Trolox equivalent. Data are mean ± SEM of three independent experiments.

**Table 3:** 2,2-Diphenyl-1-picrylhydrazyl (DPPH) of *Antirhea borbonica* plants extract and standard phenolic compounds.

	*Antirhea borbonica* (10 mM GAE)	Gallic Acid (10 mM)	Ascorbic Acid (10 mM)	Caffeic Acid (8.5 mM)
Radical scavenging capacity	82.59 ± 2.71	92.00 ± 2.54	93.20 ± 3.30	41.22 ± 1.22
(% DPPH reduced)	82.59 ± 2.71	92.00 ± 2.54	93.20 ± 3.30	41.22 ± 1.22

Free radical-scavenging activity was measured for *Ab* extract (10 mM GAE), gallic acid (10 mM), ascorbic acid (10 mM) and caffeic acid (8.5 mM) through DPPH method and expressed as % DPPH reduced. Data are mean ± SEM of three independent experiments.

**Table 4:** Impact of *Antirhea borbonica* on methylglyoxal (MGO)-induced non-glycated albumin (BSA) glycation.

	Ketoamine/BSA (mol/mol)	Fluorescent AGE (% / BSA)	Free Amine/BSA (mol/mol)	Thiols/BSA (mol/mol)	β-Amyloid formation (%/BSA)	Intrinsic Fluo Quenching (%/BSA)
**BSA**	0.07 ± 0.29	0 ± 96.38	58.86 ± 2.43	0.968 ± 0.089	0 ± 29.04	0 ± 1.54
**BSA + *Ab***	0.59 ± 0.03	97.72 ± 5.42	60.81 ± 6.20	0.178 ± 0.023 ^###^	1725.9 ± 374.7 ^###^	49.63 ± 2.38 ^###^
**BSA + MGO**	6.30 ± 0.44 ***	707.69 ± 193.15 ***	46.94 ± 1.06 **	0.384 ± 0.024 **	722.5 ± 64.9 ***	88.40 ± 0.75 ***
**BSA + MGO + *Ab***	3.97 ± 0.07 ^#^	331.80 ± 43.18 ^#^	49.55 ± 0.93 ^#^	0.426 ± 0.070	848.8 ± 198.5 ***	87.92 ± 0.29 ***

Ketoamine level was determined by using the NBT assay. Percent increases in fluorescent AGE levels were determined from the maximal fluorescence emission at an excitation wavelength of 335 nm. Unmodified primary amino group and free thiol group contents in proteins were determined by using the TNBS assay and the Ellman’s method, respectively. Percent increase in the level of β-amyloid aggregate formation probed with Thioflavin T and determined by the maximal fluorescence emission at an excitation wavelength of 450 nm. Percent decrease in intrinsic fluorescent level (quenching) obtained by the maximal fluorescence emission at an excitation wavelength of 270 nm. All data are expressed as means ± SEM of three independent experiments. *Effect of glycation (vs. BSA or vs. BSA + *Ab*), *** *p* < 0.001, ** *p* < 0.01. ^#^Effect of *Ab*) (vs. BSA or BSA + MGO), ^###^ *p* < 0.001, ^#^ *p* < 0.05.

**Table 5:** *Antirhea borbonica* extracts do not prevent hemoglobin from relative glycation in erythrocytes.

	α-Hemoglobin		β-Hemoglobin	
	∆Mass	Relative % Glycation	∆Mass	Relative % Glycation
RBC	194.0 ± 4.0	17.8 ± 0.9	188.3 ± 4.0	25.2 ± 1.1
RBC + *Ab*	187.7 ± 3.8	20.5 ± 1.2	184.0 ± 3.0	27.4 ± 0.7
RBC + MGO	194.4 ± 3.6	25.3 ± 1.8 *	192.0 ± 2.2	32.7 ± 2.9 *
RBC+MGO + *Ab*	196.3 ± 2.7	25.1 ± 1.0 ^#^	193.6 ± 1.9	33.7 ± 1.1 ^#^

Δmass and relative % glycation were calculated as explained in the methods section for RBC, RBC + *Ab*, RBC + MGO and RBC + MGO + *Ab*. Results are the mean ± SEM and statistical analyses were performed using Student’s *t*-test: * *p* < 0.05 (vs. RBC), # *p* < 0.05 (vs. RBC + *Ab*).

**Table 6:** *Antirhea borbonica* prevents MGO-induced impairments in red blood cell deformability.

	RBC	RBC + *Ab*	RBC + MGO	RBC + MGO + *Ab*
Elongation parameters				
**EI_max_**	0.428 ± 0.038	0.492 ± 0.026	0.113 ± 0.022 ***	0.239 ± 0.103 ^##^
**SS_1/2_**	4.447 ± 0.978	2.300 ± 0.090	12.259 ± 4.284 **	14.197 ± 2.765
Osmoscan parameters				
**EI_os-max_**	0.494 ± 0.066	0.495 ± 0.057	0.206 ± 0.035 **	0.302 ± 0.032 ^#^
**Ei_os-min_**	0.200 ± 0.064	0.200 ± 0.040	0.226 ± 0.055	0.192 ± 0.002
**rEI**	2.669 ± 0.660	2.573 ± 0.719	0.970 ± 0.052 *	1.572 ± 0.237

Maximum elongation index (EI_max_) and shear stress values applied at the half elongation (SS1/2) were calculated from deformability curve. Minimum elongation index (EI os-min) and maximum elongation index (EI os-max) values obtained from Osmoscan curves displayed on Figure 2A,B. The ratio of maximal and minimal EI values was calculated as follows—rEI = Ei_os-max_/Ei_os-min_. All data are expressed as mean ± SEM of at least three independent experiments. *Effect of MGO on red blood cells (vs. RBC), * *p* < 0.05, ** *p* < 0.01, *** *p* < 0.001. ^#^ Effect of *Ab* on glycated red blood cells (vs. RBC + MGO), ^#^ *p* < 0.05, ^##^ *p* < 0.01.

## Supplementary Materials

The following are available online at https://www.mdpi.com/2076-3921/9/5/415/s1, Figure S1: Experimental protocol of zebrafish experiment. Figure S2: Identification of polyphenols from *Antirhea borbonica* plant extract. Figure S3: Methylglyoxal and *Antirhea borbonica* plant extract induced change in electrophoretic pattern of albumin. Figure S4: Methylglyoxal and *Antirhea borbonica* plant extract induced albumin conformational changes. Figure S5: Characterization of glycation percentage in the different erythrocyte preparations by mass spectrometry. Figure S6: Methyl glyoxal impairs erythrocyte capacity to be deformed. Figure S7: Methyl glyoxal induced dose-dependent increase in intracellular ROS production and erythrocyte eryptosis. Figure S8: Caffeic acid induced albumin conformational changes and β-aggregation.

## References

[1] Orasanu G., Plutzky J. (2009). The pathologic continuum of diabetic vascular disease. J. Am. Coll. Cardiol..

[2] Brownlee M. (2005). The pathobiology of diabetic complications: A unifying mechanism. Diabetes.

[3] Vlassopoulos A., Lean M.E., Combet E. (2013). Role of oxidative stress in physiological albumin glycation: A neglected interaction. Free Radic. Biol. Med..

[4] Thornalley P.J., Langborg A., Minhas H.S. (1999). Formation of glyoxal, methylglyoxal and 3-deoxyglucosone in the glycation of proteins by glucose. Biochem. J..

[5] Goudarzi M., Kalantari H., Rezaei M. (2018). Glyoxal toxicity in isolated rat liver mitochondria. Hum. Exp. Toxicol..

[6] Rondeau P., Bourdon E. (2011). The glycation of albumin: Structural and functional impacts. Biochimie.

[7] Bouma B., Kroon-Batenburg L.M., Wu Y.P., Brunjes B., Posthuma G., Kranenburg O., de Groot P.G., Voest E.E., Gebbink M.F. (2003). Glycation induces formation of amyloid cross-beta structure in albumin. J. Biol. Chem..

[8] Sattarahmady N., Moosavi-Movahedi A.A., Ahmad F., Hakimelahi G.H., Habibi-Rezaei M., Saboury A.A., Sheibani N. (2007). Formation of the molten globule-like state during prolonged glycation of human serum albumin. Biochim. Biophys. Acta.

[9] Baraka-Vidot J., Guerin-Dubourg A., Bourdon E., Rondeau P. (2012). Impaired drugs-binding capacities of in vitro and in vivo glycated albumin. Biochimie.

[10] Gajahi Soudahome A., Catan A., Giraud P., Assouan Kouao S., Guerin-Dubourg A., Debussche X., Le Moullec N., Bourdon E., Bravo S.B., Paradela-Dobarro B. (2018). Glycation of human serum albumin impairs binding to the glucagon-like peptide-1 analogue liraglutide. J. Biol. Chem..

[11] Neelofar K., Ahmad J. (2017). An overview of in vitro and in vivo glycation of albumin: A potential disease marker in diabetes mellitus. Glycoconj. J..

[12] Wautier J.L., Wautier M.P., Schmidt A.M., Anderson G.M., Hori O., Zoukourian C., Capron L., Chappey O., Yan S.D., Brett J. (1994). Advanced glycation end products (AGEs) on the surface of diabetic erythrocytes bind to the vessel wall via a specific receptor inducing oxidant stress in the vasculature: A link between surface-associated AGEs and diabetic complications. Proc. Natl. Acad. Sci. USA.

[13] Babu N., Singh M. (2004). Influence of hyperglycemia on aggregation, deformability and shape parameters of erythrocytes. Clin. Hemorheol. Microcirc..

[14] Buttari B., Profumo E., Rigano R. (2015). Crosstalk between red blood cells and the immune system and its impact on atherosclerosis. Biomed. Res. Int..

[15] Catan A., Turpin C., Diotel N., Patche J., Guerin-Dubourg A., Debussche X., Bourdon E., Ah-You N., Le Moullec N., Besnard M. (2019). Aging and glycation promote erythrocyte phagocytosis by human endothelial cells: Potential impact in atherothrombosis under diabetic conditions. Atherosclerosis.

[16] Mokken F.C., Kedaria M., Henny C.P., Hardeman M.R., Gelb A.W. (1992). The clinical importance of erythrocyte deformability, a hemorrheological parameter. Ann. Hematol..

[17] Noad R.L., Rooney C., McCall D., Young I.S., McCance D., McKinley M.C., Woodside J.V., McKeown P.P. (2016). Beneficial effect of a polyphenol-rich diet on cardiovascular risk: A randomised control trial. Heart.

[18] Han X., Shen T., Lou H. (2007). Dietary Polyphenols and Their Biological Significance. Int. J. Mol. Sci..

[19] Manach C., Scalbert A., Morand C., Remesy C., Jimenez L. (2004). Polyphenols: Food sources and bioavailability. Am. J. Clin. Nutr..

[20] Giraud-Techer S., Amédé J., Girard-Valenciennes E., Thomas H., Brillant S., Grondin I., Marodon C., Smadja J. (2016). Plantes médicinales de La Réunion inscrites à la Pharmacopée française. Ethnopharmacologia.

[21] Lavergne R. (2016). Tisaneurs et Plantes Médicinales Indigenes a la Réunion.

[22] Gurib-Fakim A. (2014). Novel Plant Bioresources: Applications in Food, Medicine and Cosmetics.

[23] Smadja J., Marodon C. (2016). Le Grand Livre des Plantes Médicinales de l’ile de La Réunion: Inscrites à la Pharmacopée Française.

[24] Le Sage F., Meilhac O., Gonthier M.P. (2017). Anti-inflammatory and antioxidant effects of polyphenols extracted from *Antirhea borbonica* medicinal plant on adipocytes exposed to Porphyromonas gingivalis and Escherichia coli lipopolysaccharides. Pharmacol. Res..

[25] Marimoutou M., Le Sage F., Smadja J., Lefebvre d’Hellencourt C., Gonthier M.P., Robert-Da Silva C. (2015). Antioxidant polyphenol-rich extracts from the medicinal plants *Antirhea borbonica*, Doratoxylon apetalum and Gouania mauritiana protect 3T3-L1 preadipocytes against H_2_O_2_, TNFalpha and LPS inflammatory mediators by regulating the expression of superoxide dismutase and NF-kappaB genes. J. Inflamm..

[26] Singleton V.L., Rossi J.A. (1965). Colorimetry of Total Phenolics with Phosphomolybdic-Phosphotungstic Acid Reagents. Am. J. Enol. Vitic..

[27] Zhishen J., Mengcheng T., Jianming W. (1999). The determination of flavonoid contents in mulberry and their scavenging effects on superoxide radicals. Food Chem..

[28] Yang H., Protiva P., Cui B., Ma C., Baggett S., Hequet V., Mori S., Weinstein I.B., Kennelly E.J. (2003). New Bioactive Polyphenols from Theobroma grandiflorum (“Cupuaçu”). J. Nat. Prod..

[29] Baraka-Vidot J., Planesse C., Meilhac O., Militello V., van den Elsen J., Bourdon E., Rondeau P. (2015). Glycation alters ligand binding, enzymatic and pharmacological properties of human albumin. Biochemistry.

[30] Baraka-Vidot J., Denemont I., Ali Mcolo Z., Bourdon E., Rondeau P. (2015). Ammonium Sulfate Precipitation but not Delipidation is a Good Method for Human Albumin Preparation for Biological Studies. Int. J. Diabetes Clin. Diagn..

[31] Paradela-Dobarro B., Rodino-Janeiro B., Alonso J., Raposeiras-Roubin S., Gonzalez-Peteiro M., Gonzalez-Juanatey J., Alvarez E. (2016). Key structural and functional differences between early and advanced glycation products. J. Mol. Endocrinol..

[32] Baskurt O.K., Hardeman M.R., Uyuklu M., Ulker P., Cengiz M., Nemeth N., Shin S., Alexy T., Meiselman H.J. (2009). Parameterization of red blood cell elongation index--shear stress curves obtained by ektacytometry. Scand. J. Clin. Lab. Investig..

[33] Nemeth N., Kiss F., Miszti-Blasius K. (2015). Interpretation of osmotic gradient ektacytometry (osmoscan) data: A comparative study for methodological standards. Scand. J. Clin. Lab. Investig..

[34] Arias C.F., Arias C.F. (2017). How do red blood cells know when to die?. R. Soc. Open Sci..

[35] Howe K., Clark M.D., Torroja C.F., Torrance J., Berthelot C., Muffato M., Collins J.E., Humphray S., McLaren K., Matthews L. (2013). The zebrafish reference genome sequence and its relationship to the human genome. Nature.

[36] Dorsemans A.C., Soule S., Weger M., Bourdon E., Lefebvre d’Hellencourt C., Meilhac O., Diotel N. (2017). Impaired constitutive and regenerative neurogenesis in adult hyperglycemic zebrafish. J. Comp. Neurol..

[37] Intine R.V., Olsen A.S., Sarras M.P. (2013). A zebrafish model of diabetes mellitus and metabolic memory. J. Vis. Exp..

[38] Singh V.P., Bali A., Singh N., Jaggi A.S. (2014). Advanced glycation end products and diabetic complications. Korean J. Physiol. Pharmacol..

[39] Kattoor A.J., Pothineni N.V.K., Palagiri D., Mehta J.L. (2017). Oxidative Stress in Atherosclerosis. Curr. Atheroscler. Rep..

[40] Senoner T., Dichtl W. (2019). Oxidative Stress in Cardiovascular Diseases: Still a Therapeutic Target?. Nutrients.

[41] Ahn S.M., Byun K., Cho K., Kim J.Y., Yoo J.S., Kim D., Paek S.H., Kim S.U., Simpson R.J., Lee B. (2008). Human microglial cells synthesize albumin in brain. PLoS ONE.

[42] Machado A.P., Pinto R.S., Moyses Z.P., Nakandakare E.R., Quintao E.C., Passarelli M. (2006). Aminoguanidine and metformin prevent the reduced rate of HDL-mediated cell cholesterol efflux induced by formation of advanced glycation end products. Int. J. Biochem. Cell Biol..

[43] Scalbert A., Williamson G. (2000). Dietary Intake and Bioavailability of Polyphenols. J. Nutr..

[44] Danino O., Gottlieb H.E., Grossman S., Bergman M. (2009). Antioxidant activity of 1,3-dicaffeoylquinic acid isolated from Inula viscosa. Food Res. Int..

[45] McCarty M.F. (2005). A chlorogenic acid-induced increase in GLP-1 production may mediate the impact of heavy coffee consumption on diabetes risk. Med. Hypotheses.

[46] Gordon M.H., Wishart K. (2010). Effects of chlorogenic acid and bovine serum albumin on the oxidative stability of low density lipoproteins in vitro. J. Agric. Food. Chem..

[47] Mullen W., Boitier A., Stewart A.J., Crozier A. (2004). Flavonoid metabolites in human plasma and urine after the consumption of red onions: Analysis by liquid chromatography with photodiode array and full scan tandem mass spectrometric detection. J. Chromatogr. A.

[48] Stalmach A., Mullen W., Barron D., Uchida K., Yokota T., Cavin C., Steiling H., Williamson G., Crozier A. (2009). Metabolite profiling of hydroxycinnamate derivatives in plasma and urine after the ingestion of coffee by humans: Identification of biomarkers of coffee consumption. Drug Metab. Dispos..

[49] Rondeau P., Navarra G., Cacciabaudo F., Leone M., Bourdon E., Militello V. (2010). Thermal aggregation of glycated bovine serum albumin. Biochim. Biophys. Acta Proteins Proteom..

[50] Kang J., Liu Y., Xie M.-X., Li S., Jiang M., Wang Y.-D. (2004). Interactions of human serum albumin with chlorogenic acid and ferulic acid. Biochim. Biophys. Acta Gen. Subj..

[51] Tang D., Li H.-J., Li P., Wen X.-D., Qian Z.-M. (2008). Interaction of Bioactive Components Caffeoylquinic Acid Derivatives in Chinese Medicines with Bovine Serum Albumin. Chem. Pharm. Bull..

[52] Li X., Chen D., Wang G., Lu Y. (2013). Study of interaction between human serum albumin and three antioxidants: Ascorbic acid, a-tocopherol and proanthocyanidins. Eur. J. Med. Chem..

[53] Lo C.-Y., Hsiao W.-T., Chen X.-Y. (2011). Efficiency of Trapping Methylglyoxal by Phenols and Phenolic Acids. J. Food Sci..

[54] Tagliazucchi D., Martini S., Conte A. (2019). Protocatechuic and 3,4-Dihydroxyphenylacetic Acids Inhibit Protein Glycation by Binding Lysine through a Metal-Catalyzed Oxidative Mechanism. J. Agric. Food Chem..

[55] Bourdon E., Loreau N., Blache D. (1999). Glucose and free radicals impair the antioxidant properties of serum albumin. FASEB J..

[56] Tupe R.S., Sankhe N.M., Shaikh S.A., Phatak D.V., Parikh J.U., Khaire A.A., Kemse N.G. (2015). Aqueous extract of some indigenous medicinal plants inhibits glycation at multiple stages and protects erythrocytes from oxidative damage-an in vitro study. J. Food Sci. Technol..

[57] Kiruthiga P.V., Shafreen R.B., Pandian S.K., Devi K.P. (2007). Silymarin protection against major reactive oxygen species released by environmental toxins: Exogenous H_2_O_2_ exposure in erythrocytes. Basic Clin. Pharmacol. Toxicol..

[58] Kiruthiga P.V., Shafreen R.B., Pandian S.K., Arun S., Govindu S., Devi K.P. (2007). Protective effect of silymarin on erythrocyte haemolysate against benzo(a)pyrene and exogenous reactive oxygen species (H2O2) induced oxidative stress. Chemosphere.

[59] Konyalioglu S., Karamenderes C. (2005). The protective effects of Achillea L. species native in Turkey against H(2)O(2)-induced oxidative damage in human erythrocytes and leucocytes. J. Ethnopharmacol..

[60] Asgary S., Naderi G., Askari N. (2005). Protective effect of flavonoids against red blood cell hemolysis by free radicals. Exp. Clin. Cardiol..

[61] Lee P., Wu X. (2015). Review: Modifications of human serum albumin and their binding effect. Curr. Pharm. Des..

[62] Smith A.S., Nowak R.B., Zhou S., Giannetto M., Gokhin D.S., Papoin J., Ghiran I.C., Blanc L., Wan J., Fowler V.M. (2018). Myosin IIA interacts with the spectrin-actin membrane skeleton to control red blood cell membrane curvature and deformability. Proc. Natl. Acad. Sci. USA.

[63] Paiva-Martins F., Fernandes J., Rocha S., Nascimento H., Vitorino R., Amado F., Borges F., Belo L., Santos-Silva A. (2009). Effects of olive oil polyphenols on erythrocyte oxidative damage. Mol. Nutr. Food Res..

[64] Paiva-Martins F., Fernandes J., Santos V., Silva L., Borges F., Rocha S., Belo L., Santos-Silva A. (2010). Powerful protective role of 3,4-dihydroxyphenylethanol-elenolic acid dialdehyde against erythrocyte oxidative-induced hemolysis. J. Agric. Food Chem..

[65] Paiva-Martins F., Goncalves P., Borges J.E., Przybylska D., Ibba F., Fernandes J., Santos-Silva A. (2015). Effects of the olive oil phenol metabolite 3,4-DHPEA-EDAH2 on human erythrocyte oxidative damage. Food Funct..

[66] Heckler K., Kroll J. (2017). Zebrafish as a Model for the Study of Microvascular Complications of Diabetes and Their Mechanisms. Int. J. Mol. Sci..

[67] Jorgens K., Stoll S.J., Pohl J., Fleming T.H., Sticht C., Nawroth P.P., Hammes H.P., Kroll J. (2015). High tissue glucose alters intersomitic blood vessels in zebrafish via methylglyoxal targeting the VEGF receptor signaling cascade. Diabetes.

[68] Rastegar S., Parimisetty A., Cassam Sulliman N., Narra S.S., Weber S., Rastegar M., Viranaicken W., Couret D., Planesse C., Strahle U. (2019). Expression of adiponectin receptors in the brain of adult zebrafish and mouse: Links with neurogenic niches and brain repair. J. Comp. Neurol..

[69] Patche J., Girard D., Catan A., Boyer F., Dobi A., Planesse C., Diotel N., Guerin-Dubourg A., Baret P., Bravo S.B. (2017). Diabetes-induced hepatic oxidative stress: A new pathogenic role for glycated albumin. Free Radic. Biol. Med..

