# Supplementary material for: Antirhea borbonica Aqueous Extract Protects Albumin and Erythrocytes from Glycoxidative Damages
Source: Antioxidants (Basel). 2020 May 12;9(5):415. doi: 10.3390/antiox9050415 (PMC7278591; doi:10.3390/antiox9050415)
Supplement: Supplementary file 1 [file antioxidants-09-00415-s001.pdf]

Supplementary materials

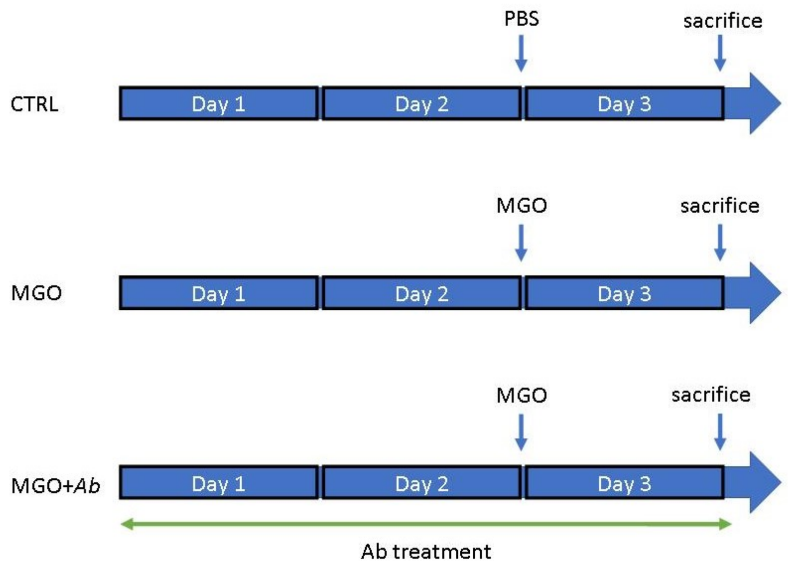

Figure S1: Experimental protocol for zebrafish experiments. Zebrafishes were divided into three groups: (CTRL) control fish treated with PBS, (MGO) fish treated with MGO (100 mg/kg of body weight) and (MGO+*Ab*) fish treated with MGO (100 mg/kg of body weight) and one more day *Ab*.

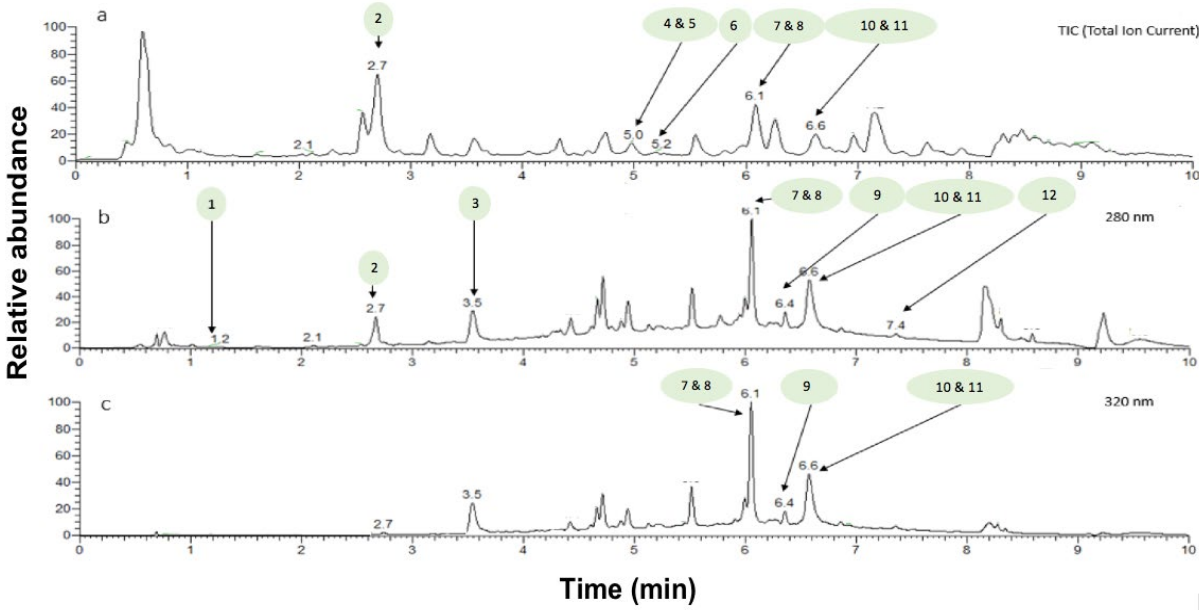

Figure S2: Identification of polyphenols from *A. borbonica* plant extract. Polyphenol-rich plant extracts were analysed by using a Q Exactive Plus mass spectrometer. Compounds were identified according to their retention time (min)/molecular weight (Da).

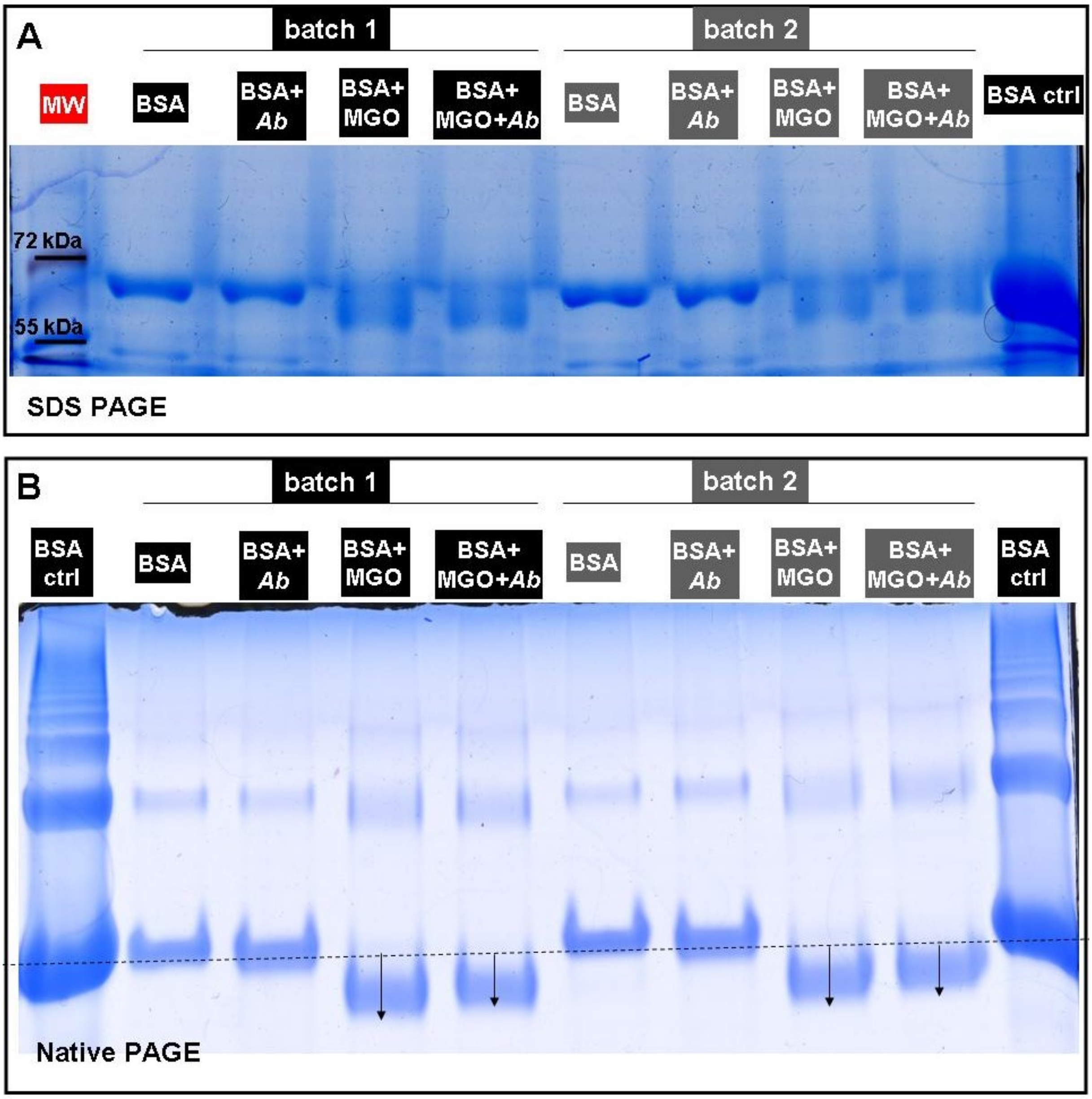

Figure S3: Methylglyoxal and *A. borbonica* plant extract induced change in electrophoretic pattern of albumin. A) Coomassie blue staining of albumin samples (5 µg, for two different batches) separated by sodium dodecyl sulphate (SDS)-polyacrylamide gel electrophoresis (PAGE) (12.5%); B) electrophoretic migration profile of native polyacrylamide gel electrophoresis (12.5%); BSA ctrl (60 µg) correspond to native albumin (without any incubation at 37°C).

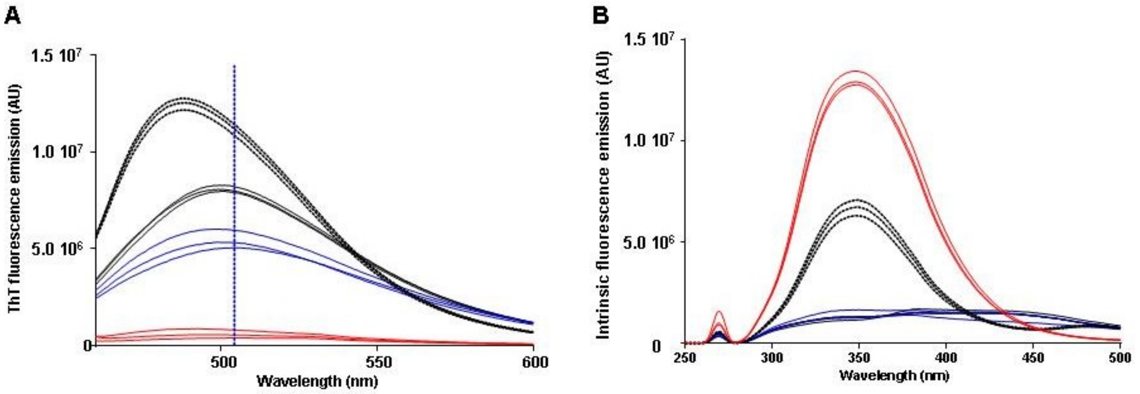

Figure S4: Methylglyoxal and *A. borbonica* plant extract induced albumin conformational changes. A) Normalized fluorescence spectra of Thioflavin T (ThT) at 435 nm wavelength excitation; **B**) Normalized intrinsic fluorescence spectra (tryptophan and tyrosine) at 270 nm excitation wavelength for BSA (red line), BSA+*Ab* (black dotted line), AGE (blue line) and AGE+*Ab* (black plain line).

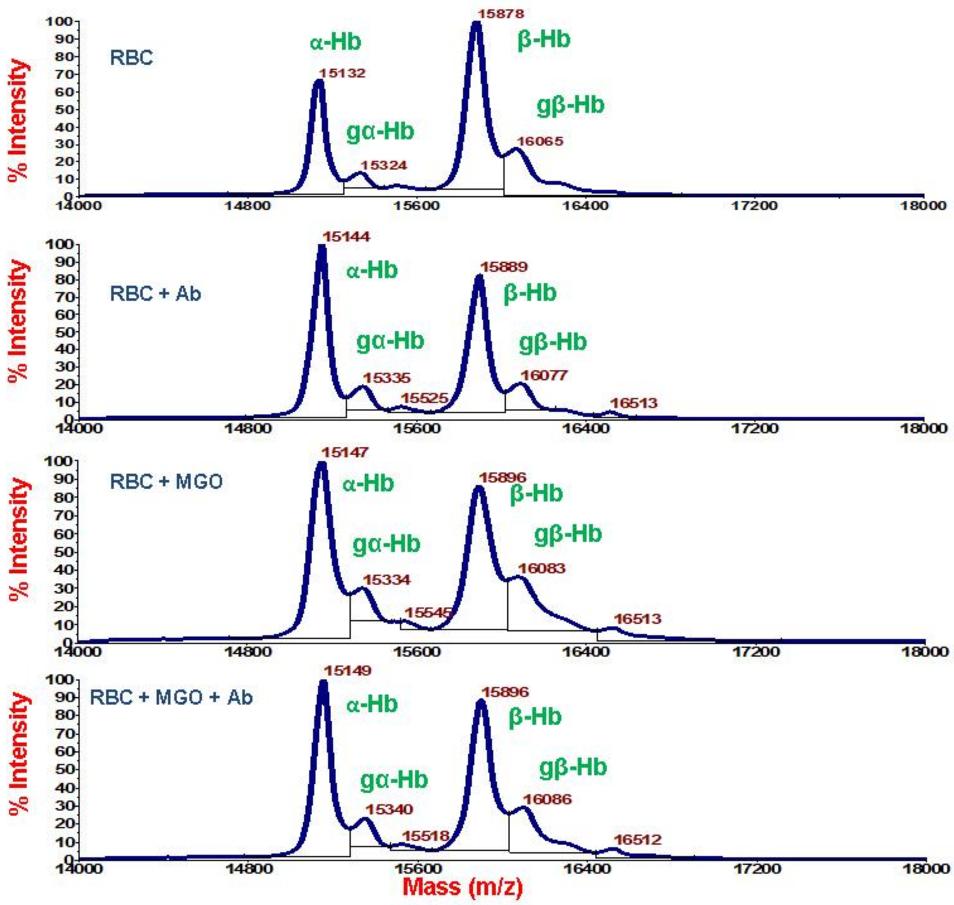

Figure S5: Characterisation of glycation percentage in the different erythrocyte preparations by mass spectrometry.

Representative figures of the mass spectra obtained in three independent experiments for each incubation condition: RBC, RBC+*Ab*, RBC+MGO and RBC+MGO+*Ab*. On each spectrum, four main peaks were obtained corresponding to α-hemoglobin (α–Hb; 15140 Da), glycated α-hemoglobin (gα–Hb; 15333 Da), β-hemoglobin (β-Hb; 15890 Da), glycated β-hemoglobin (gβ-Hb; 16080 Da).

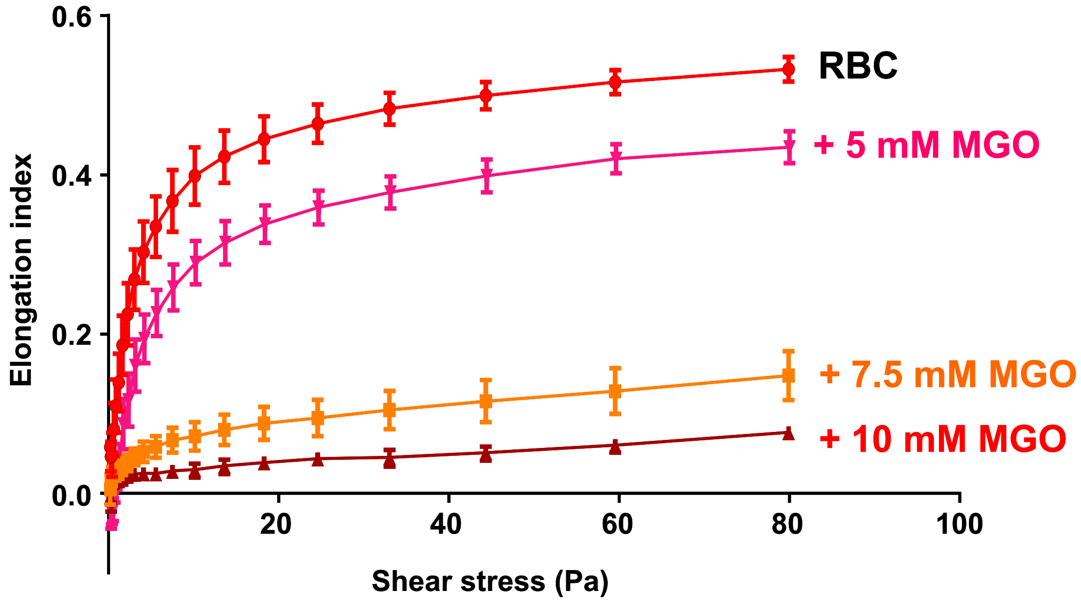

Figure S6: Methyl glyoxal impairs erythrocyte capacity to be deformed. Curves represent erythrocyte deformation as elongation index as a function of shear stress for RBC (red line); RBC+5 mM MGO (pink line); RBC+ 7.5 mM MGO (orange line) and RBC+ 10 mM MGO (brown line) erythrocytes samples. Data are mean ± SEM of four independent experiments.

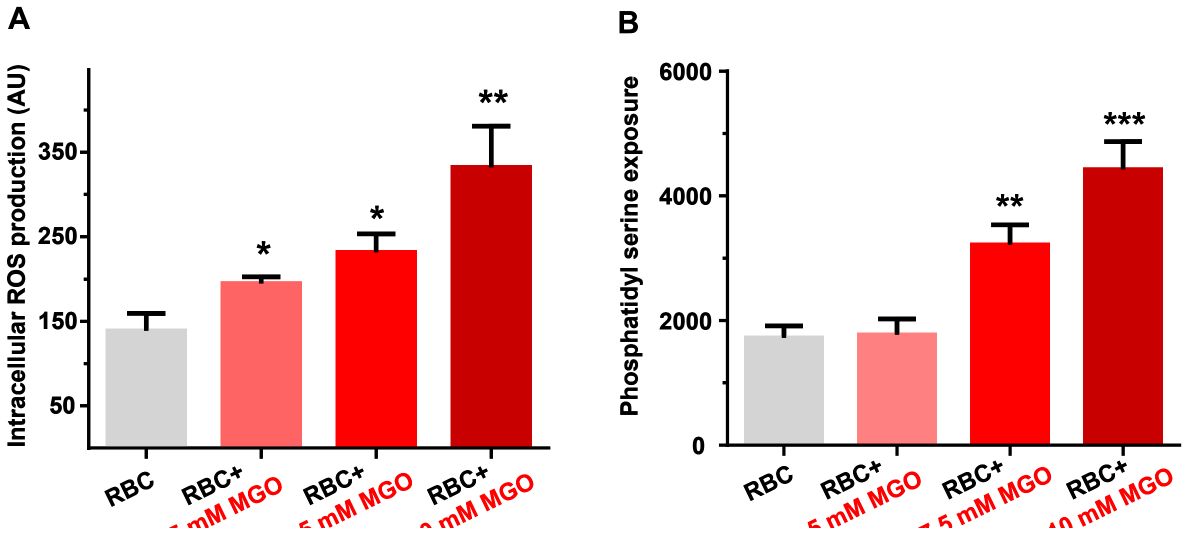

Figure S7: Methyl glyoxal induced dose-dependent increase in intracellular ROS production and erythrocyte eryptosis. A) Intracellular ROS levels were measured by flow cytometry using HDCF-DA fluorescent probe and **B)** phosphatidylserine exposure (PS) was determined with annexin V-FITC fluorescent probes. Data are mean ± SEM of four independent experiments. *Effect of MGO (*vs.* RBC), $^{***}p<0.001$, $^{**}p<0.01$, $^{*}p<0.05$.

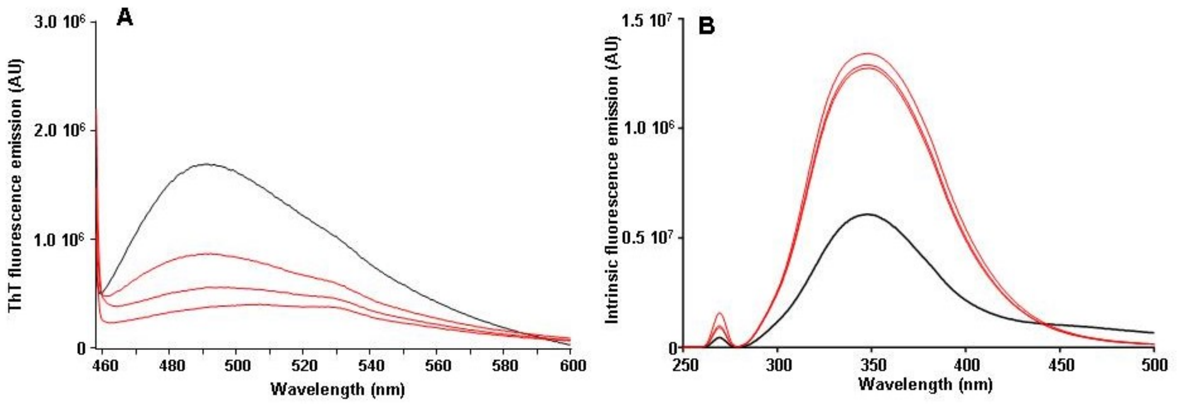

Figure S8: Caffeic acid induced albumin conformational changes and β-aggregation. A) Normalized fluorescence spectra of Thioflavin T (ThT) at 435 nm wavelength excitation and specific probe for β-aggregates; **B**) Normalized intrinsic fluorescence spectra at 270 nm excitation wavelength for BSA (red line) (performed in triplicate), BSA+Caffeic acid (black line). BSA (20 g/L in PBS) was incubated during 7 days in the absence or presence of caffeic acid (360 μM final concentration).
